# Supplementary figures and images for: NOX5-induced uncoupling of endothelial NO synthase is a causal mechanism and theragnostic target of an age-related hypertension endotype
Source: PLoS Biol. 2020 Nov 10;18(11):e3000885. doi: 10.1371/journal.pbio.3000885 (PMC7654809; doi:10.1371/journal.pbio.3000885)

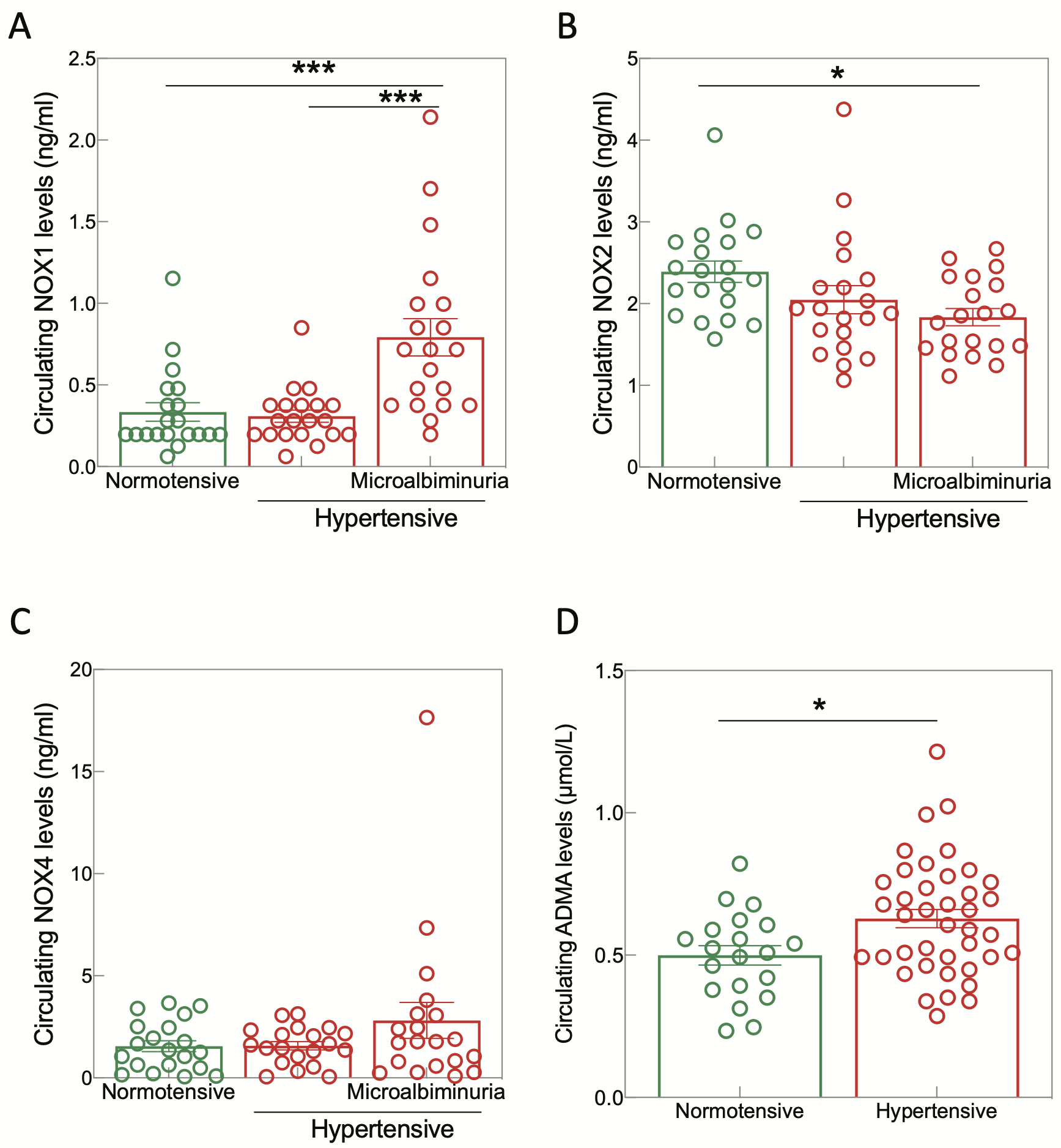

Supplement: S1 Fig — (A–C) There were no differences in NOX1, NOX2, or NOX4 levels between hypertensive patients with normoalbuminuria (n = 20) compared to normotensive individuals (n = 20). NOX1 levels were higher (A), but NOX2 were lower (B) in hypertensive patients with microalbuminuria (n = 20) compared to normotensive individuals. Comparison between groups was done by one-way ANOVA followed by Tukey’s multiple comparisons test. (D) ADMA levels were significantly higher in hypertensive patients (n = 40) compared to healthy individuals (n = 20). Comparison between the two groups was done by two-tailed unpaired t test. All data are represented as mean ± SEM, *p < 0.05, ***p < 0.001. All raw data are included in the S1 Data file. (TIF) [file pbio.3000885.s001.tif]

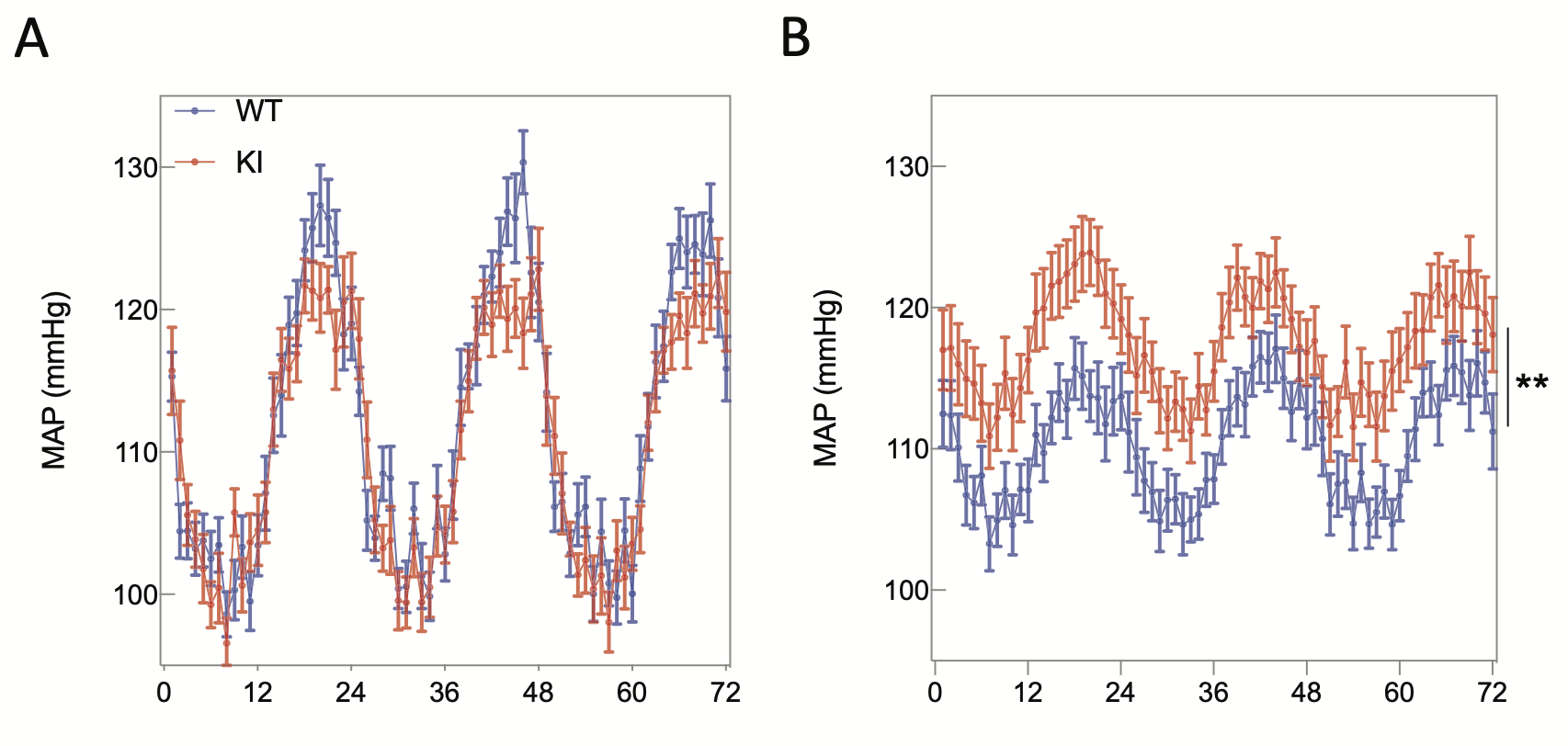

Supplement: S2 Fig — (A) There was no significant difference in MAP between young WT (n = 19) and KI (n = 20). (B) Aged KI mice (n = 33) had higher MAP compared to WT (n = 31). Telemetry data were analyzed by two-way repeated measures ANOVA followed by Sidak’s multiple comparisons test. All data are represented as mean ± SEM of n individual animals **p < 0.01. All raw data are included in the S1 Data file. (TIF) [file pbio.3000885.s002.tif]

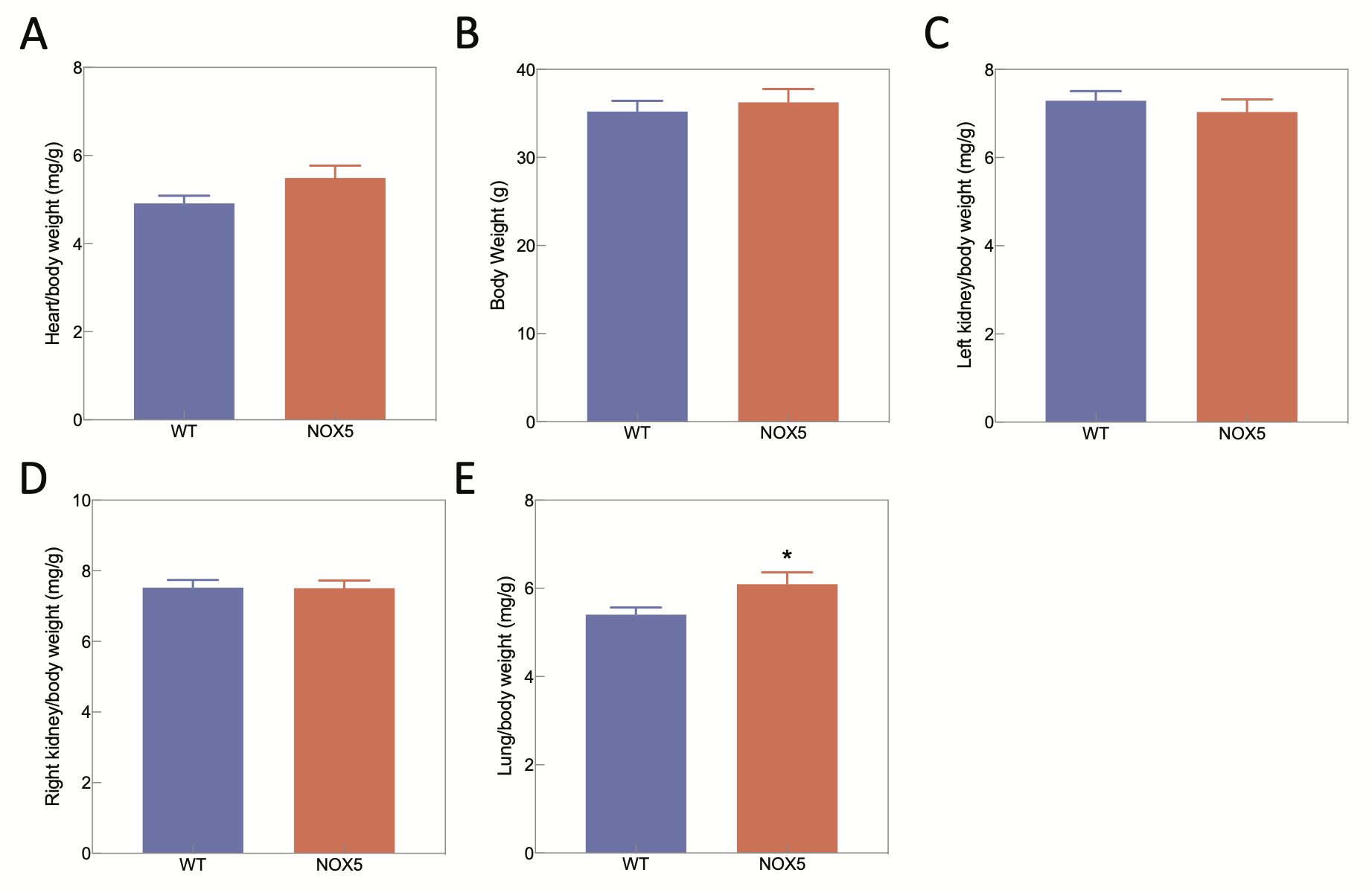

Supplement: S3 Fig — (A–E) There was no difference in body, heart, and kidney weights between WT (n = 24) and KI mice (n = 20); however, lung/body weight ratio was higher in KI mice. Comparison between groups were done by two-tailed unpaired t test. All data are represented as mean ± SEM of n individual animals *p < 0.05. (TIF) [file pbio.3000885.s003.tif]

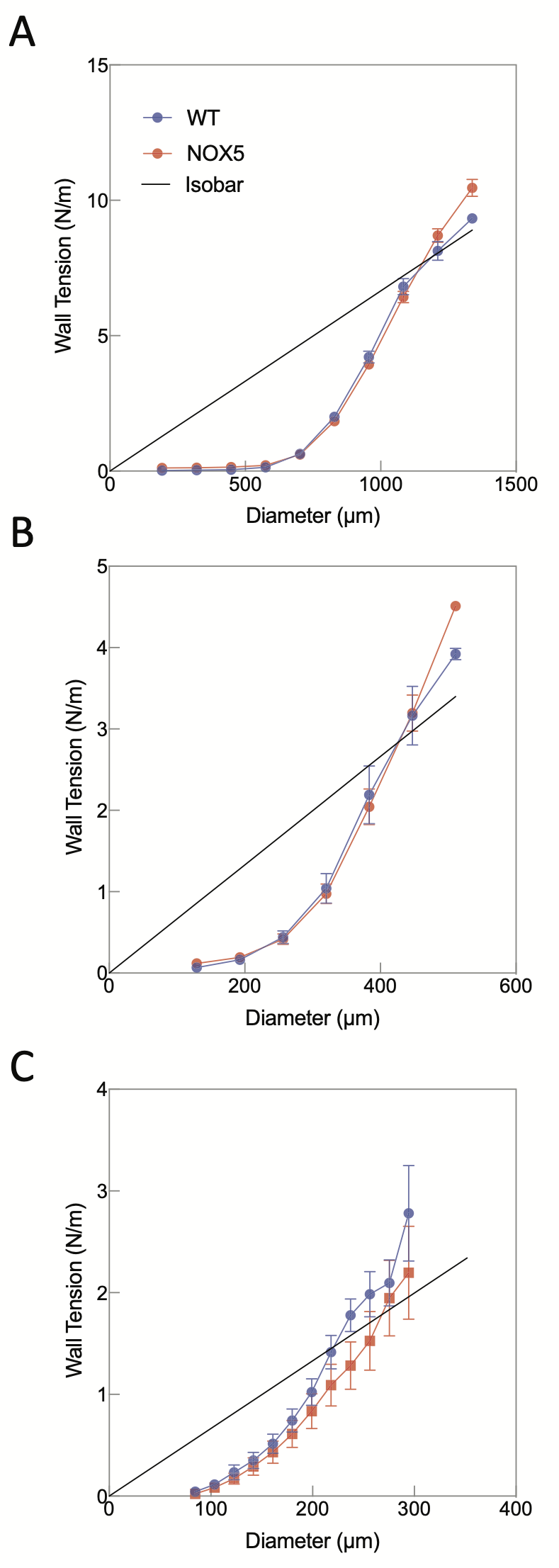

Supplement: S4 Fig — (A–C) The relation between resting wall tension and arterial lumen diameter did not differ between KI (n = 9) and WT (n = 9) mice in thoracic aortae (A), femoral arteries (B), and saphenous arteries (C). All data are represented as mean ± SEM of n individual animals. All raw data are included in the S1 Data file. (TIF) [file pbio.3000885.s004.tif]

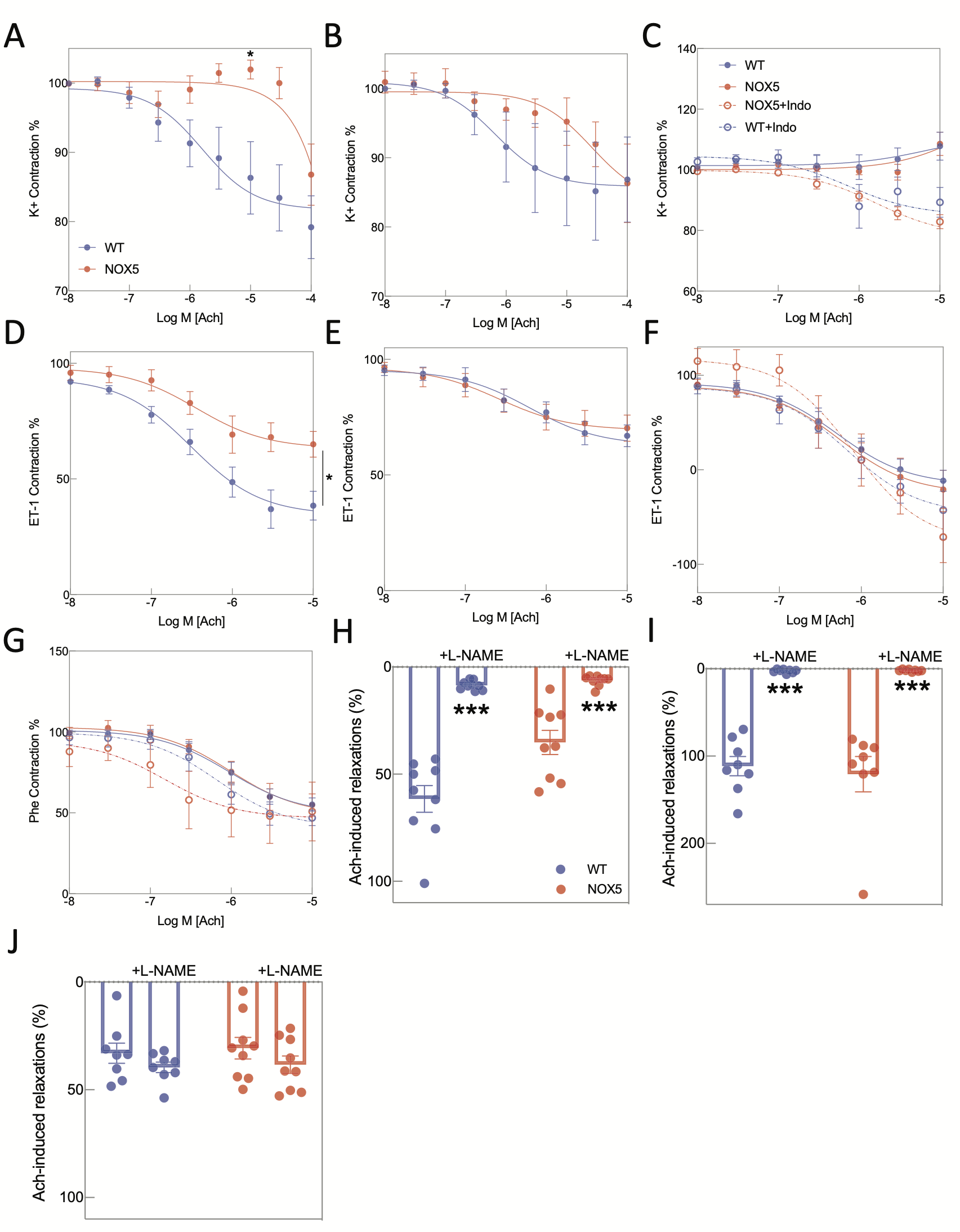

Supplement: S5 Fig — (A–C) Ach-induced relaxations were impaired in femoral arteries (A) of aged KI mice (n = 9) compared to WT (n = 8–9) but not in saphenous arteries (B) and thoracic aortae (with/without indomethacin) (C) precontracted with K+. (D–F) Ach-induced relaxations were impaired in femoral arteries (D) of aged KI mice (n = 9) compared to WT (n = 8–9) but not in saphenous arteries (E) and thoracic aortae with/without indomethacin (F) precontracted with endothelin-1. (G) There was no difference in Ach-induced relaxations in thoracic aortae (with/without indomethacin) precontracted with phenylephrine between WT (n = 9) and KI mice (n = 9). (H–J) Ach-induced relaxations in arteries made to contract with endothelin-1 were reversed by 100 μM L-NAME in femoral arteries (H) and thoracic aortae (I), but not saphenous arteries (J) of both aged KI mice (n = 8–9) and WT (n = 8–9). Myograph data were analyzed by two-way ANOVA followed by Sidak’s multiple comparisons test. All data are represented as mean ± SEM of n individual animals, *p < 0.05. All raw data are included in the S1 Data file. (TIF) [file pbio.3000885.s005.tif]

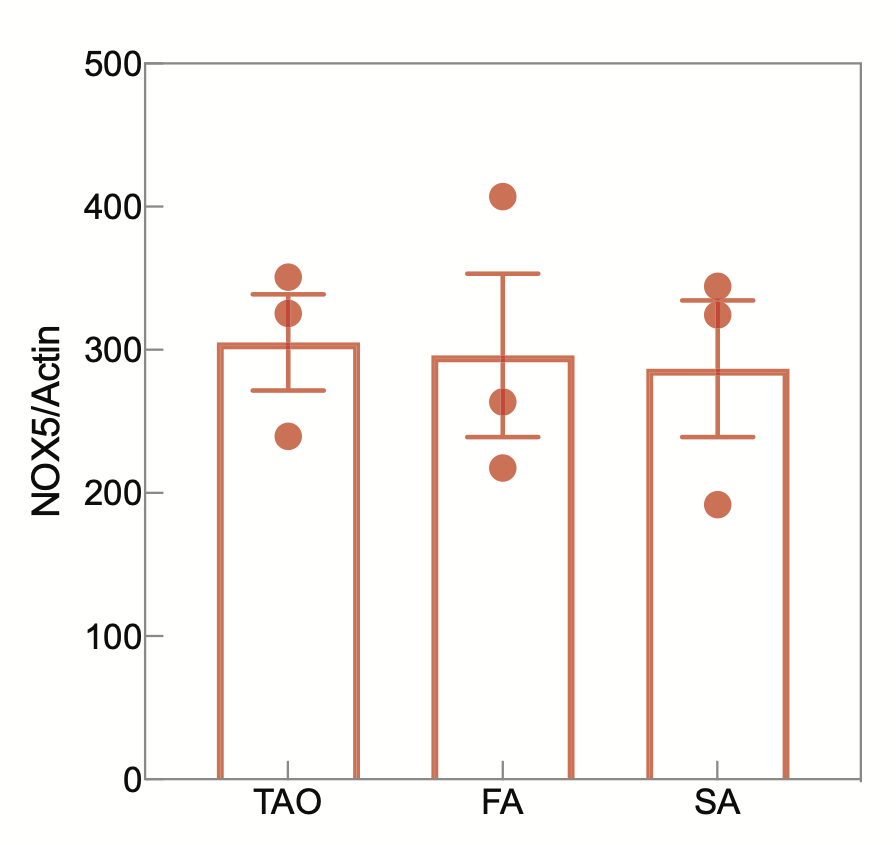

Supplement: S6 Fig — There was no difference in NOX5 gene expression between the 3 vessel types (n = 3, each in duplicates). Comparison were done by one-way ANOVA. All data are represented as mean ± SEM of n individual animals. All raw data are included in the S1 Data file. FA, femoral artery; SA, saphenous artery; TAO, thoracic aortae. (TIF) [file pbio.3000885.s006.tif]

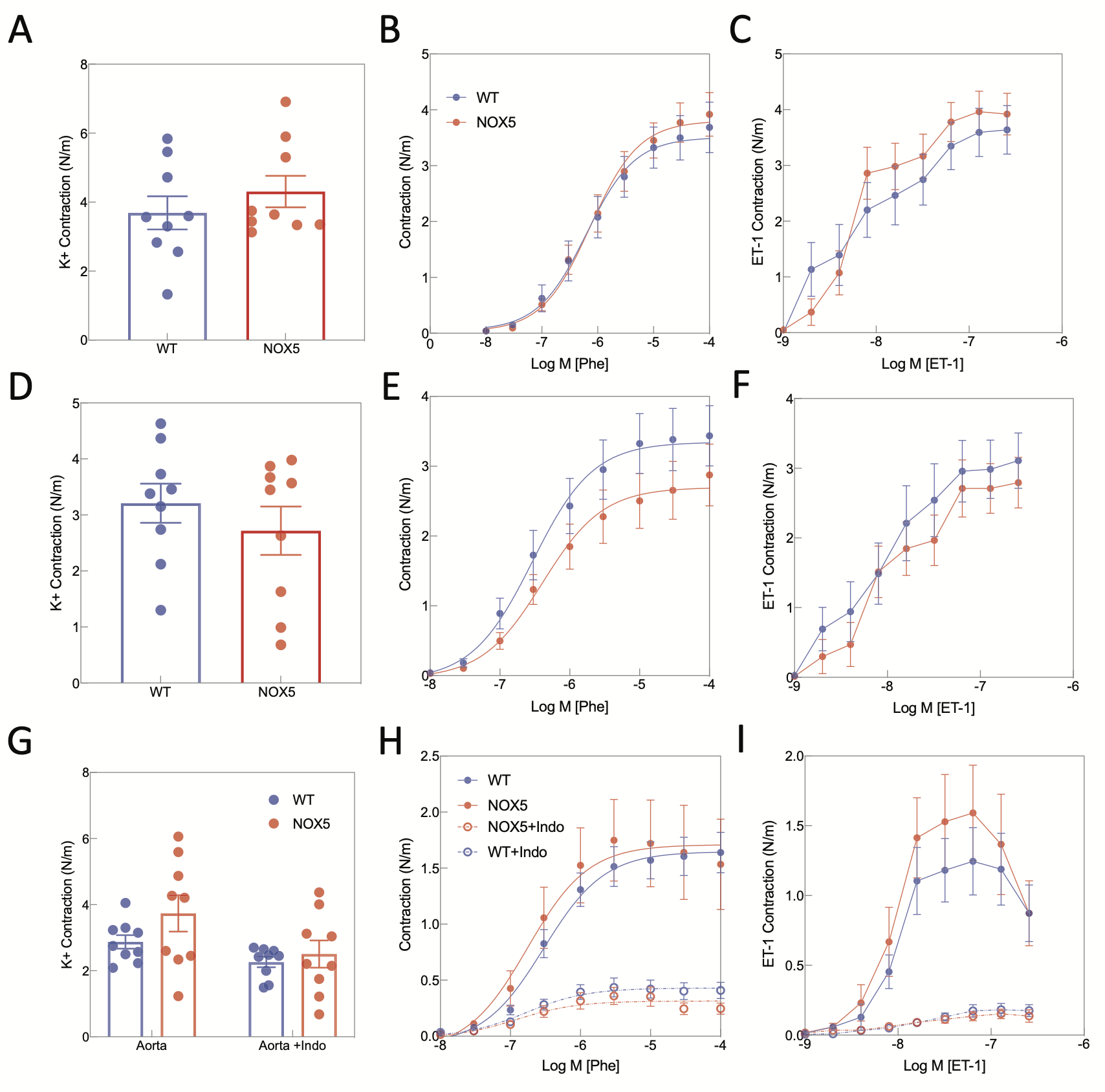

Supplement: S7 Fig — There was no difference in contractile responses to K+, phenylephrine, and endothelin-1 in femoral arteries (A–C), saphenous arteries (D–F), and thoracic aortae (with/without indomethacin) (G–I) between WT (n = 8–9) and KI mice (n = 9). Comparison between 2 groups in contractile responses to K+ was done by two-tailed t test. Other myograph data were analyzed by two-way ANOVA followed by Sidak’s multiple comparisons test. All data are represented as mean ± SEM of n individual animals. All raw data are included in the S1 Data file. (TIF) [file pbio.3000885.s007.tif]

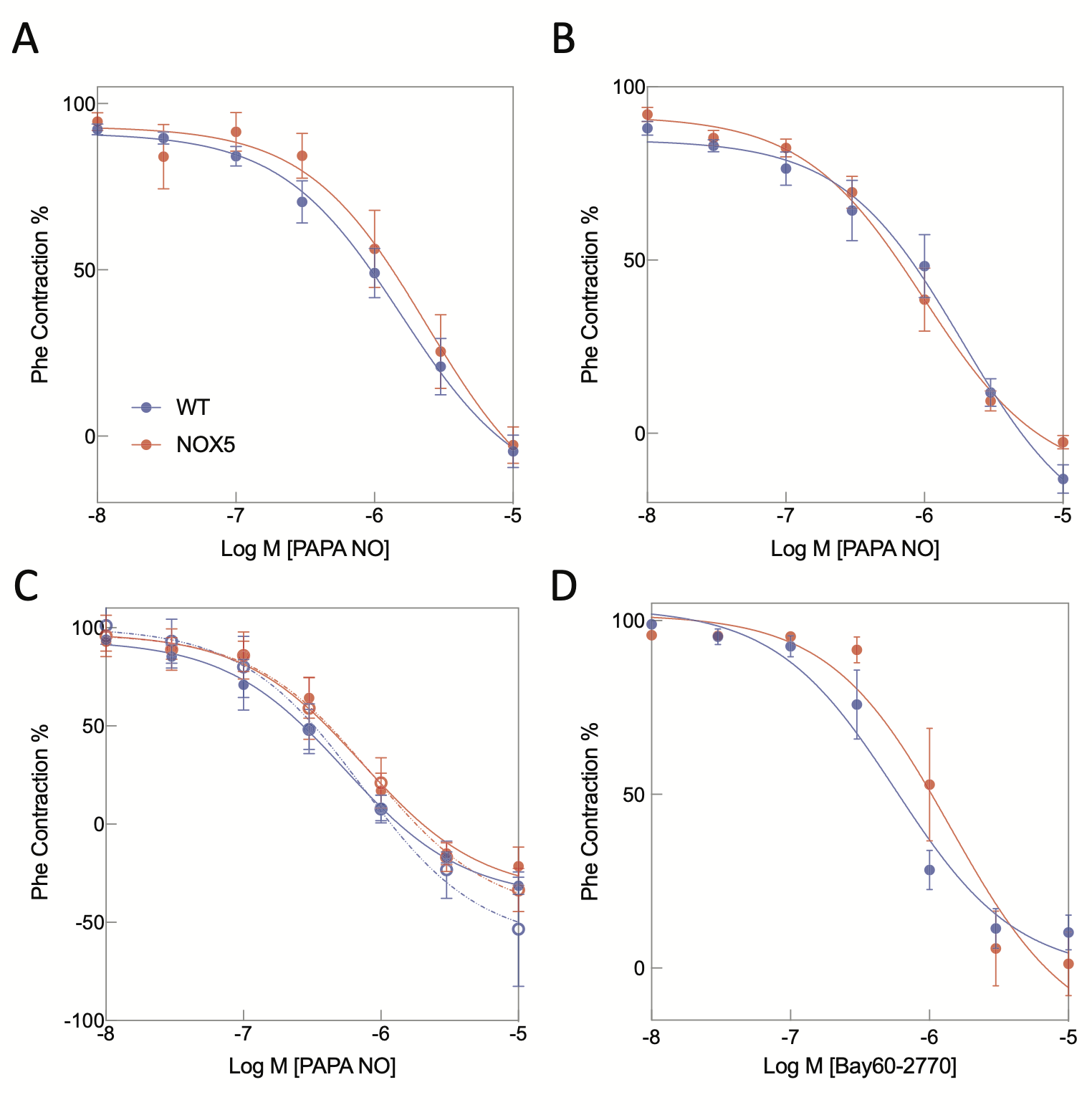

Supplement: S8 Fig — (A–C) Relaxations induced by the NO donor, PAPA NO (0.01–10 μM), in femoral arteries (A), saphenous arteries (B), and thoracic aortae (with/without indomethacin) (C) did not differ between WT (n = 8–9) and KI mice (n = 9). (D) Relaxations induced by the apo-sGC activator, Bay60-2770 (0.01–10 μM), in femoral arteries did not differ between WT (n = 4) and KI mice (n = 4). Myograph data were analyzed by two-way ANOVA followed by Sidak’s multiple comparisons test. All data are represented as mean ± SEM of n individual animals. All raw data are included in the S1 Data file. (TIF) [file pbio.3000885.s008.tif]

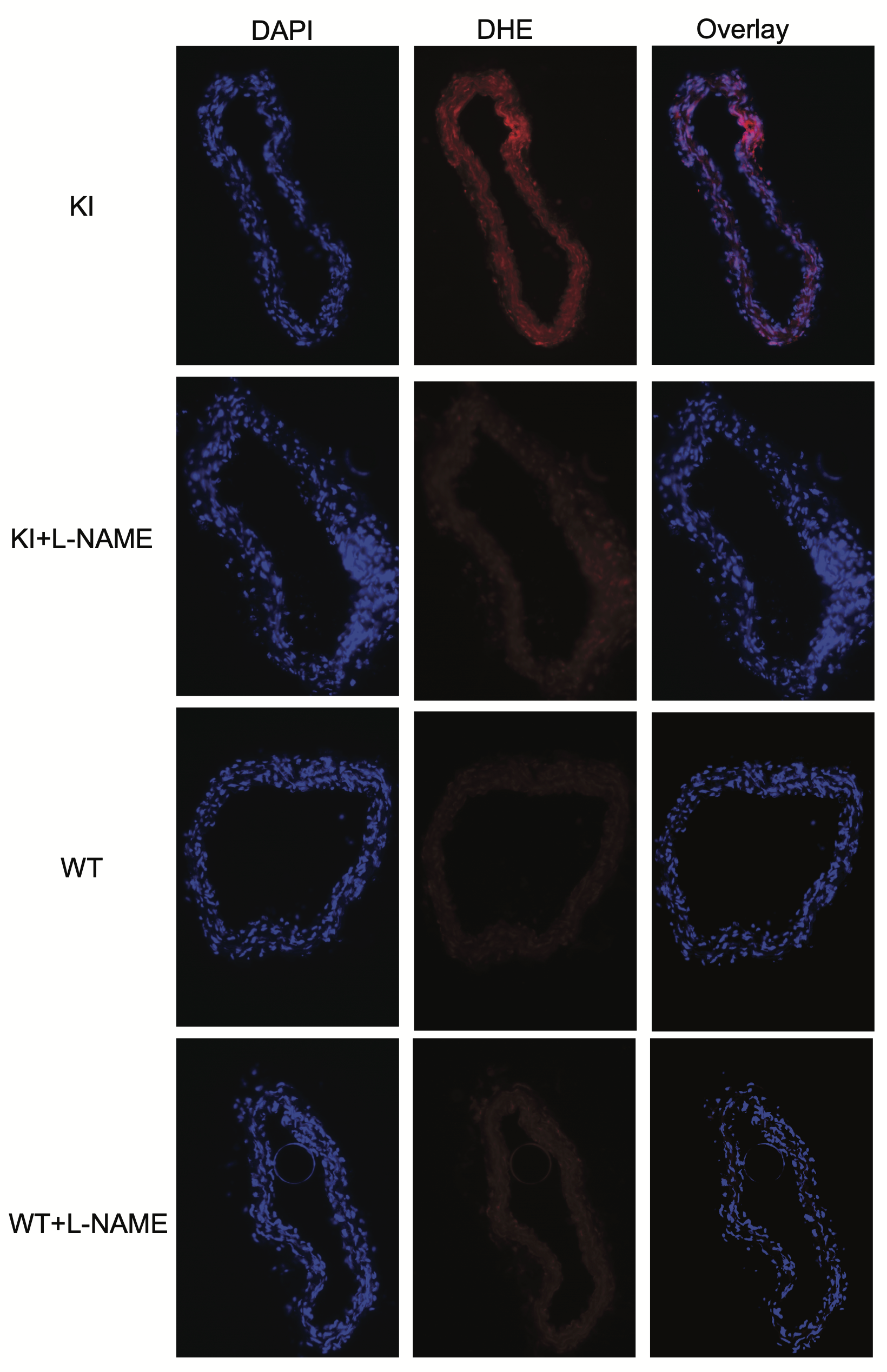

Supplement: S9 Fig — (TIF) [file pbio.3000885.s009.tif]

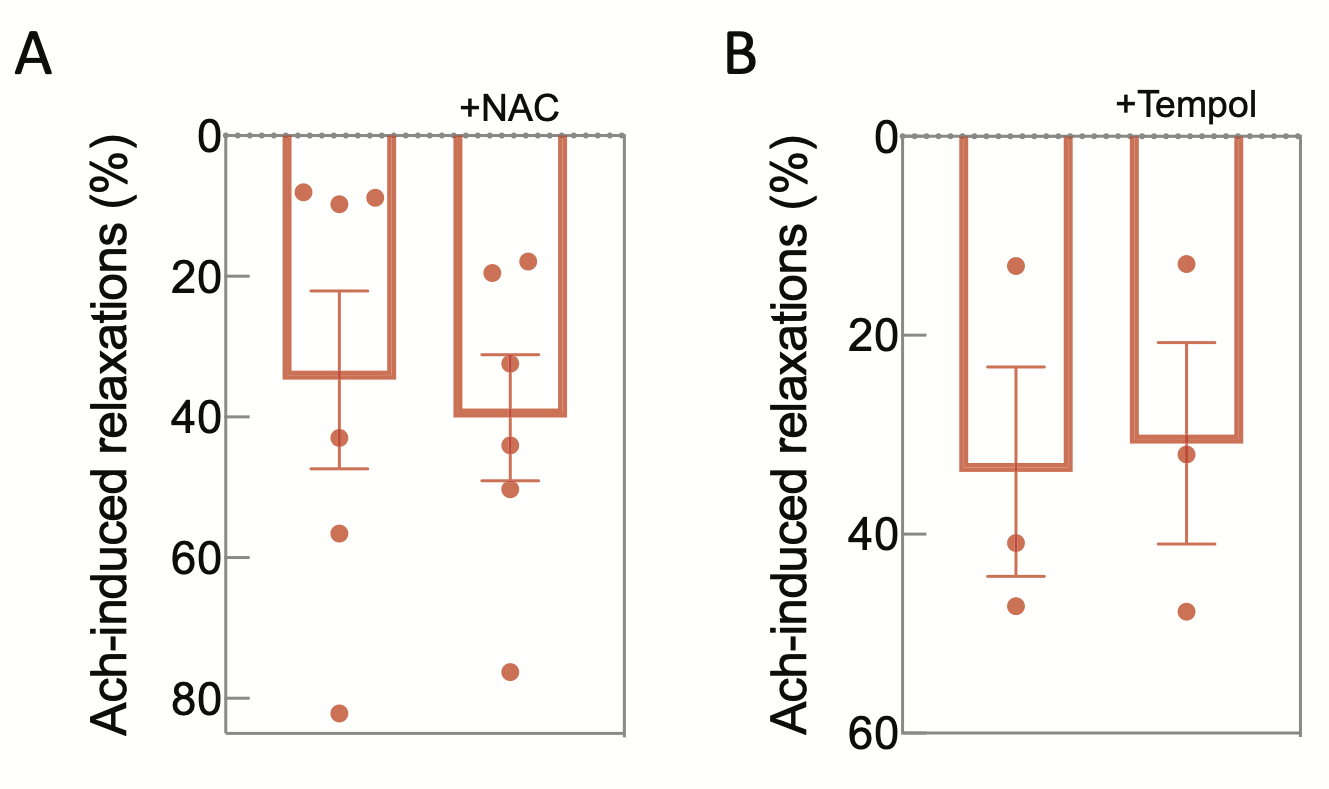

Supplement: S10 Fig — In segments of femoral artery (n = 3–6) made to contract with 10 μM phenylephrine, relaxing effects of Ach (10 μM) were not reversed by 10 μM N-acetylcysteine (NAC) (A) or 100 μM tempol (B). Comparison between groups were done by two-tailed unpaired t test. All data are represented as mean ± SEM of n individual animals. All raw data are included in the S1 Data file. (TIF) [file pbio.3000885.s010.tif]

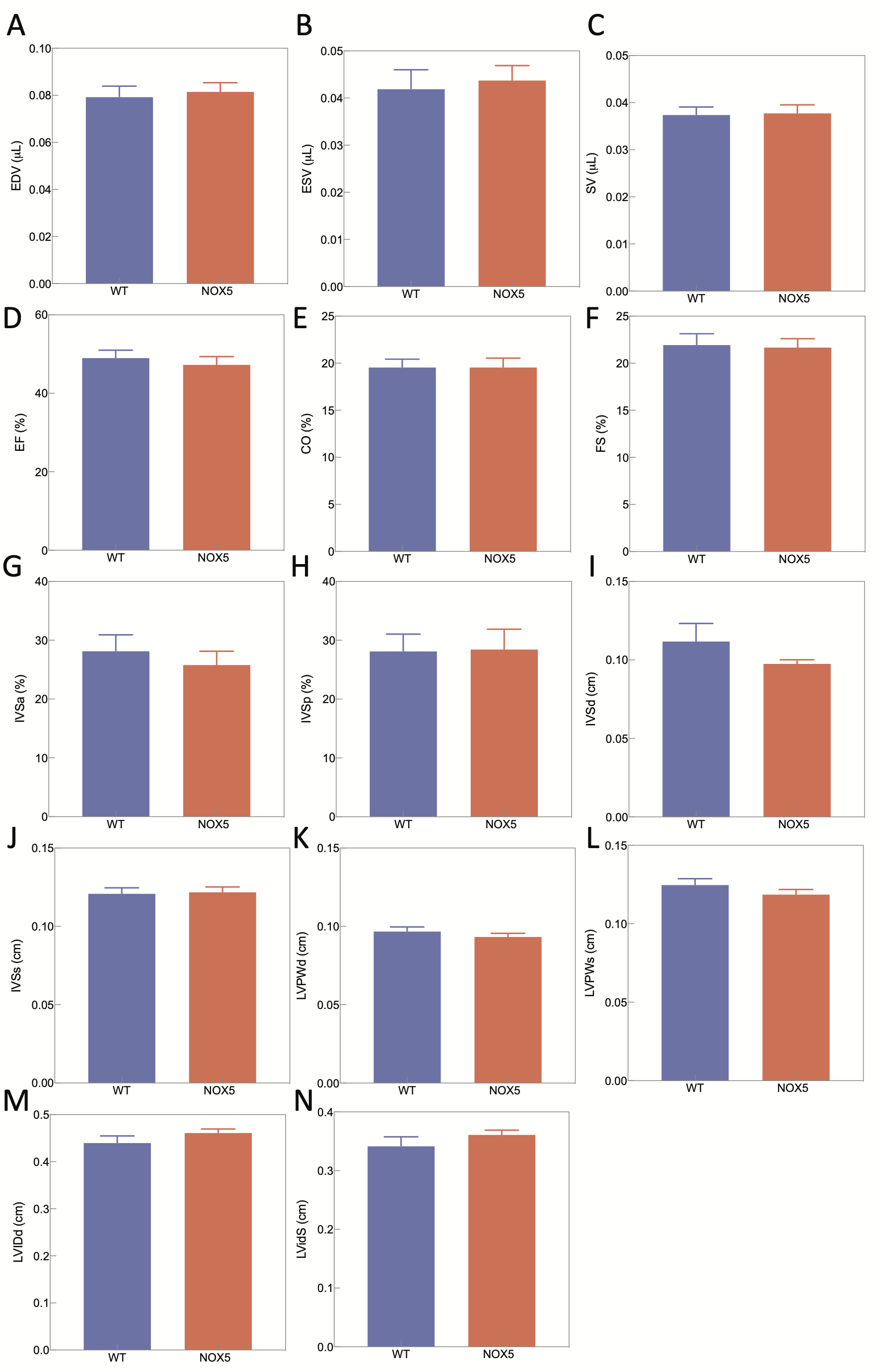

Supplement: S11 Fig — There were no differences in all parameters between WT (n = 28) and KI mice (n = 29). Comparison between groups were done by two-tailed unpaired t test. All data are represented as mean ± SEM of n individual animals. All raw data are included in the S1 Data file. (TIF) [file pbio.3000885.s011.tif]

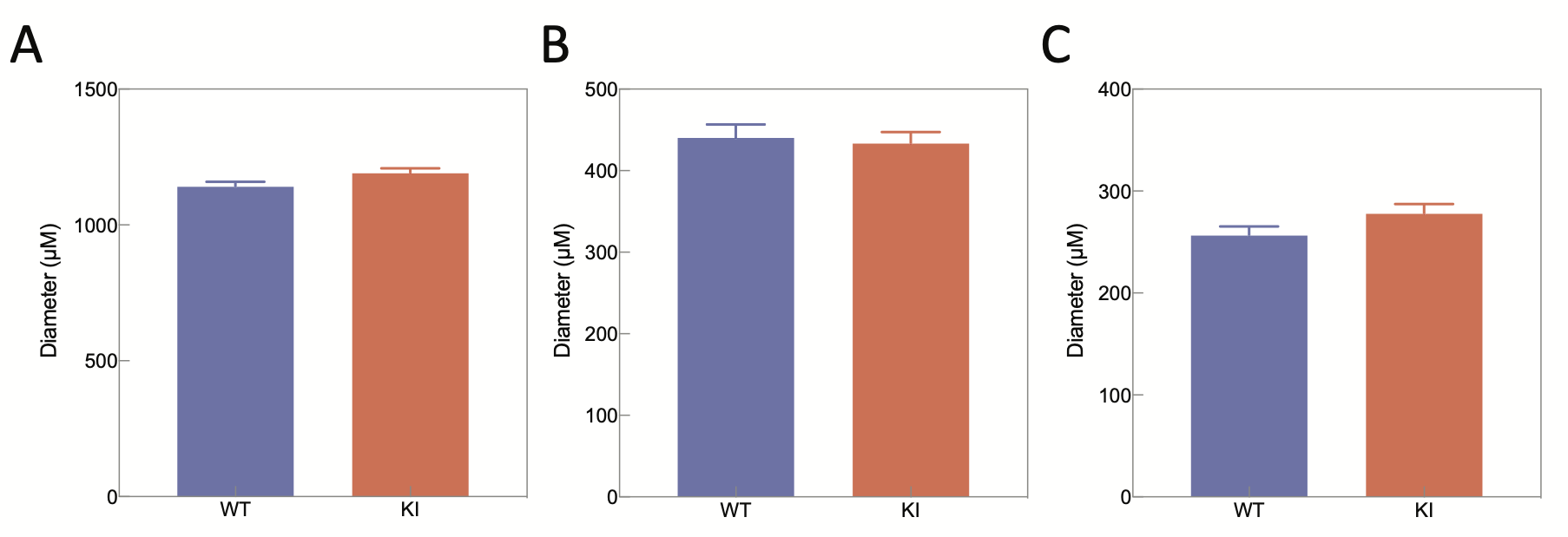

Supplement: S12 Fig — There were no differences in diameter of thoracic aortae (A), femoral arteries (B), and saphenous arteries (with/without indomethacin) between WT (n = 9) and KI mice (n = 9). Comparison between groups were done by two-tailed unpaired t test. All data are represented as mean ± SEM of n individual animals. All raw data are included in the S1 Data file. (TIF) [file pbio.3000885.s012.tif]
