# Supplementary material for: NOX5-induced uncoupling of endothelial NO synthase is a causal mechanism and theragnostic target of an age-related hypertension endotype
Source: PLoS Biol. 2020 Nov 10;18(11):e3000885. doi: 10.1371/journal.pbio.3000885 (PMC7654809; doi:10.1371/journal.pbio.3000885)
Supplement: Attachment — Submitted filename: Appeal rebuttal letter.pdf [file pbio.3000885.s016.pdf]

To
Di Jiang
Editor, PLOS Biology

Your ref.	*Our ref.*	*Direct line*	*Maastricht*
		+31 43 38-81421	2019-02-06

RE NOX5-induced uncoupling of endothelial NO synthase is a causal mechanism and theragnostic target of an age-related hypertension endotype

Dear Dr. Jiang,

Thank you for considering our manuscript No. PBIOLOGY-D-20-00252, entitled "NOX5-induced uncoupling of endothelial NO synthase is a causal mechanism and theragnostic target of an age-related hypertension endotype". Unfortunately, you felt it lacks strength on the advance as NOX5 has a known roles in other models related to hypertension such as stroke and myocardial infarction (MI). this is not the case and the manuscripts represents a breakthrough in our approach to and understanding of hypertension that is independent of its possible complications, stroke and MI, and which is rapidly translated into a multinational clinical trial.

1. The role of NOX5 in myocardial infarction is not at all clear. There is only correlation between expression of NOX5 in blood vessels and cardiomyocytes and acute myocardial infarction (Hahn et al., 2012). In addition, when we checked this possible causality we rejected this hypothesis as we did not find a role for endothelial NOX5 in MI in our previous publication (Casas et al., 2019).

2. The role of NOX5 in stroke that we showed in our publication (Casas et al., 2019) is entirely unrelated to hypertension but a reperfusion phenomenon due to a surge in calcium influx that can be observed in young NOX5 KI mice that are normotensive. The main role in stroke is due to NOX4, which we had earlier published in a very successful paper in PLoS Biology (Kleinschnitz et al., 2010) and later one in PNAS (Casas et al., 2017).

We show for the first time a mechanism-based endotype of hypertension, which is currently treated only symptomatically with vasodilatory compounds and can now be treated for a subgroup of approximately 25% in a mechanism-based manner.

We clearly identify NOX5 as the causal agent within the disease module of this endotype and we show the most likely mechanism, i.e. NOS uncoupling, and provide a possible therapeutic approach, i.e. NOS recoupling with sepiapterin.

Aged NOX5 KI mice are thus the only known mechanism-based model of human hypertension, i.e. a model that does not just mimic the symptom, elevated blood pressure, but also the cause for it.

Finally our manuscript shows a clinical diagnostic and stratification approach by measuring highly elevated NOX5 levels in liquid biopsies.

Given these arguments, we trust that you will agree now that our m/s has substantial strength on the advances with respect to NOX5 and hypertension in general and would thus appreciate if you honored our commitment to PLoS Biology for our best work by considering our manuscript for peer review.

References

Casas, A. I., Geuss, E., Kleikers, P. W. M., Mencl, S., Herrmann, A. M., Buendia, I., . . . Schmidt, H. (2017). NOX4-dependent neuronal autotoxicity and BBB breakdown explain the superior sensitivity of the brain to ischemic damage. *Proc Natl Acad Sci U S A*. doi:10.1073/pnas.1705034114

Casas, A. I., Kleikers, P. W., Geuss, E., Langhauser, F., Adler, T., Busch, D. H., . . . Schmidt, H. H. (2019). Calcium-dependent blood-brain barrier breakdown by NOX5 limits postreperfusion benefit in stroke. *J Clin Invest, 130*, 1772-1778. doi:10.1172/JCI124283

Hahn, N. E., Meischl, C., Kawahara, T., Musters, R. J., Verhoef, V. M., van der Velden, J., . . . Krijnen, P. A. (2012). NOX5 expression is increased in intramyocardial blood vessels and cardiomyocytes after acute myocardial infarction in humans. *Am J Pathol, 180*(6), 2222-2229. doi:10.1016/j.ajpath.2012.02.018

Kleinschnitz, C., Grund, H., Wingler, K., Armitage, M. E., Jones, E., Mittal, M., . . . Schmidt, H. H. (2010). Post-stroke inhibition of induced NADPH oxidase type 4 prevents oxidative stress and neurodegeneration. *PLoS biology, 8*(9). doi:10.1371/journal.pbio.1000479

Thank you in advance.

Sincerely,

Harald H.H.W. Schmidt, MD, PhD
Dept. Pharmacology and Personalised Medicine
Faculty of Health, Medicine & Life Sciences

Mahmoud Elbatreek, PharmD
