# Supplementary material for: NOX5-induced uncoupling of endothelial NO synthase is a causal mechanism and theragnostic target of an age-related hypertension endotype
Source: PLoS Biol. 2020 Nov 10;18(11):e3000885. doi: 10.1371/journal.pbio.3000885 (PMC7654809; doi:10.1371/journal.pbio.3000885)
Supplement: Attachment — Submitted filename: NOX5_Response to Editor and Reviewers comments_v8.pdf [file pbio.3000885.s017.pdf]

To
Di Jiang
Editor
PLOS Biology

Your ref.	*Our ref.*	*Direct line*	*Maastricht*
		+31433881421	2020-05-31

RE NOX5-induced uncoupling of endothelial NO synthase is a causal mechanism and theragnostic target of an age-related hypertension endotype (PBIOLOGY-D-20-00252R1)

Dear Dr. Jiang,

Thank you and the reviewers for the valuable comments on our manuscript entitled **“NOX5-induced uncoupling of endothelial NO synthase is a causal mechanism and theragnostic target of an age-related hypertension endotype”**.

Please find attached our revised manuscript in which we were able to address all points raised by the reviewers. We have updated the introduction, results, figures, discussion and methods. In addition, we have added new data and their relevant figures.

As recommended by the first reviewer, some statements have been clarified in the introduction, some results have been extensively explained and new data regarding levels of other NOXs in human samples, NOS uncoupling in NOX5KI mice and some *ex vivo* experiments have been added.

As recommended by the second reviewer, telemetry-related figures have been updated and mean arterial pressure data has been included.

As recommended by the third reviewer, the discussion has been updated with more balanced interpretations and declaration of some limitations in our study in addition new data regarding testing antioxidants in the *ex vivo* experiments have been included and discussed.

As recommended by the fourth reviewer, figures are now presented in a higher resolution. For details, we attach **a point-by-point response to every reviewer’s comments.**

Visiting address
Universiteitssingel 50
6229 ER Maastricht
Postal address
P.O. Box 616
6200 MD Maastricht
The Netherlands

T +31 43 388 1421
F +31 43 388 4149
ING Bank: 065.76.25.418
IBAN: NL47 INGB 065.76.25.418
BIC: INGBNL2A

Our main findings and conclusions remain unchanged: we here report to our knowledge the first causal molecular mechanism of human systolic hypertension associated with ageing. This endotype affects approximately one in four patients and molecularly consists of a disease module of NOX5-induced uncoupling of endothelial NO synthase followed by impaired endothelium-dependent vasodilation in muscular conduit arteries.

Detection of elevated levels of NOX5 in circulating microparticles appears to serve as a mechanism-based liquid biopsy marker to stratify patients for therapeutic intervention. Based on our in-vivo validation in aged NOX5 mice and tissue such an intervention may include the tetrahydrobiopterin precursor and NOS recoupling agent, sepiapterin, and, once such compounds become clinically available, NOX5 inhibitors.

We therefore trust that you will find our revised manuscript suitable to be considered for publication in PLoS Biology.

Sincerely,

Harald Schmidt, MD PhD

Mahmoud Elbatreek, PharmD

Point-by-point response to the reviewers

Reviewer 1

Comment 1: In this study, mice with Nox5 knocked in were studied and found to have hypertension with age. Some evidence for NOS uncoupling was shown. There are multiple questions that arise upon reading the manuscript which detract.

Response: We thank the reviewer for the constructive criticisms and valuable comments. We thoroughly addressed all of the reviewer's comments/questions throughout the entire manuscript.

Comment 2: Your statement in the intro regarding renal artery stenosis as the only cause of hypertension with a known mechanism is wrong. Other known causes include adrenal adenomas, pheochromocytomas and numerous single gene mutations involving renal transporters.

Response: We agree with the reviewer that other causes of secondary hypertension were missing in the manuscript, therefore, we have added them to the revised version of the manuscript. Those include adrenal adenomas, pheochromocytomas and numerous single gene mutations involving renal transporters in addition to renal artery stenosis [1]. This is now added to the manuscript (page 5, line 74-77).

Comment 3: The statement: " . . . antihypertensive therapy is neither curative nor effective, needing high numbers to treat with many patients still experiencing adverse outcomes" doesn't make sense. Do you mean that therapy requires high doses of drugs or several agents to achieve target pressures?

Response: Current antihypertensive therapy focuses on treating the symptom i.e. the high blood pressure and not the underlying causal mechanisms. Even in some cases, the symptom is not treated i.e. treatment-resistant hypertension. In addition, antihypertensives have high numbers to treat which means that many patients will still experience adverse outcomes such as stroke and myocardial infarction despite being treated with these drugs. We have clarified this in the manuscript (page 5, line 78-81).

Comment 4: The machine learning/prunned network analysis is only hypothesis generating. An important experimental finding is that Nox5 is highly expressed in human microparticles. It is essential that the investigators show that there is not an increase in other Nox isoforms in these microparticles.

Response: We have measured the levels of other vascular NOX isoforms (NOX1, NOX2 and NOX4) in samples of normotensive and hypertensives patients (with and without microalbuminuria). There were no significant differences in all the three NOXs levels between normotensive and hypertensive patients. However, patients with microalbuminuria showed higher NOX1, but lower NOX2 levels compared to normotensive subjects (see S1A-C Figure and also below). Indeed, previous preclinical studies showed that NOX1 does not play a role in hypertension, but with renal oxidative stress [2] and renal pathology [3-5]. Endothelial NOX2 also does not contribute to the induction of hypertension without infusion of angiotensin II [6, 7]. Endothelial NOX4 is rather vasoprotective [8] and its deletion has no effect on blood pressure [9]. This has been addressed in the manuscript (page 5,9, line 90-94, 193-199).

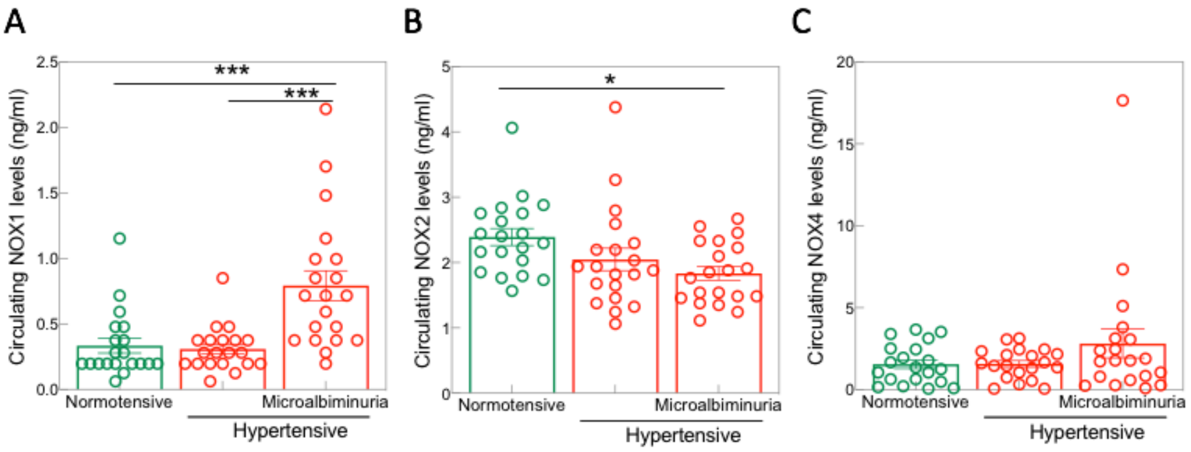

Comment 5: It seems odd that there is no cardiac hypertrophy in the Nox5 KI mice given that they are hypertensive.

Response: That the trend for cardiac hypertrophy in the NOX5 KI mice did not yet reach statistical significance, is in line with the late (with ageing) development of their hypertension in response to alterations in their muscular conduit arteries. In addition, cardiac hypertrophy does not necessarily exist with hypertension. For example, other hypertensive animal models such as eNOS KO mice do not have cardiac hypertrophy [10, 11]. This is also in agreement with clinical observations that not all hypertensive patients (subgroups) have cardiac hypertrophy [12-14]. We have discussed this in the manuscript (page11, line 228-231).

Comment 6: It is unclear how the diameter tension relationships were constructed in figure S2. It seems these are just tensions at different degrees of stretch in the wire myograph. This is a very crude way to determine stress strain relationships, which should be analyzed in pressurized vascular segments as described by Humphrey and co-workers (PMID: 23549898). It is very unusual that there is not vascular stiffening in the Nox5 mice, if they are really hypertensive.

Response: The diameter tension relationships were constructed according to the law of Laplace for a cylindrical tube: P = T/R (with P for transmural pressure, T for wall tension and R for the lumen radius of the tube). Consequently, wall stiffness can be determined equally by recording 1) changes in tension in response to imposed changes in radius (wire myography) or 2) changes in radius in response to changes in transmural pressure (pressure myography). One of the coauthors (Jo De Mey) previously used both approaches interchangeably during several decades (approx. 15 papers between the following references [15, 16]). Here we used the former approach because it has a higher throughput than the latter. Stress-strain relationships and even better "incremental elastic (Young's) modulus" (which require additional recording of wall thickness) only come into play to discriminate between structural and material property contributions to changes in arterial stiffness. Contrary to the last statement of the reviewer, structural stiffening of arteries is not a general observation in rodent models of (essential) hypertension [17-19]. The scientific literature in this respect was among others reviewed by Integan and Schiffrin [20]. Also, clinical data especially from aged hypertensive patients agrees with this [21-24]. This has been adapted in the revised version of the manuscript (page 12 and 27, line 258-261 and 607-616).

Comment 7: The evidence that sepiapterin improves endothelium-dependent vasodilatation is only partial support that NOS uncoupling has occurred. Can the authors show an increase in vascular superoxide production that is inhibited by L-NAME? Can they demonstrate that the NOS3 enzyme migrates as a monomer on low temperature gel? Would treatment with sepiapterin lower blood pressure?

Response: We provide new data showing that superoxide is increased (as indicated by DHE staining) in femoral arteries of NOX5KI mice and this is inhibited by L-NAME pretreatment (see S9 Figure and also below). These data have been added to the revised manuscript in the methods and results (page 15

and 27-28, line 333-335 and 623-633). With respect to NOS3 monomerization on gel, we believe that this is irrelevant to NOS uncoupling as recently shown [25]. Regarding treatment of mice with sepiapterin, unfortunately, it cannot be addressed experimentally in a reasonable period of time. The elevation of systolic BP in NOX5 KI mice became apparent when we recorded hemodynamics in mice of progressively increasing age. These mice had to be euthanized in order to isolate their blood vessels and test hypotheses concerning the underlying mechanisms. The size of the colonies of mice is small and it would take many months/years to reach the necessary number of animals of advanced age to do the necessary experiments. This experiment would have strengthened our conclusion about NOX5-induced NOS uncoupling. Indeed, sepiapterin or BH4 were shown in some preclinical and clinical studies to slightly reduce the high blood pressure induced by NOS uncoupling [26-28]. We have included a statement in the "discussion" of our manuscript that refers to this desirable extension of our mechanistic *ex vivo* analysis of impaired endothelium-dependent relaxation (uncoupled eNOS) through this additional set of therapeutic *in vivo* experiments (recoupling eNOS; see page 19, line 436-438).

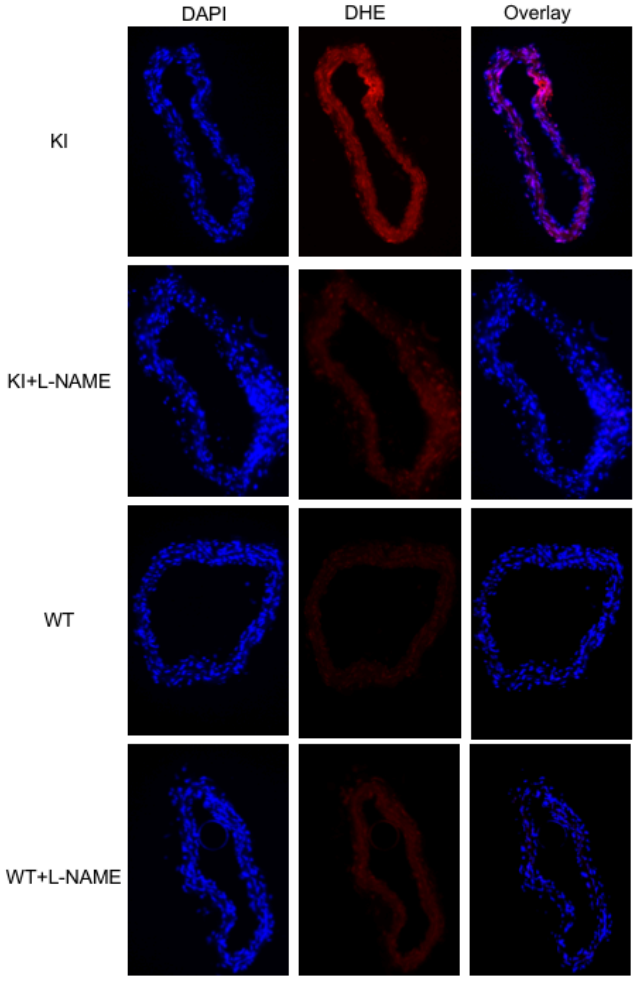

<u>Comment 8</u>: The differences in endothelium-dependent vasodilatation between the femoral artery (affected) and the saphenous artery and aorta (not affected) is hard to understand. It is known that branch vessels are less dependent on NO, but this should affect the saphenous and femoral arteries similarly.

<u>Response:</u> In this study and in earlier studies of our group [29-31], Ach-induced relaxations of saphenous arteries of the mouse are largely resistant to pharmacological inhibition of NOS and display many characteristics of being mediated by endothelium-dependent hyperpolarization. On the other hand, in their muscular conduit femoral artery (the continuation of the iliac artery in the hindleg) made to contract with ET-1, the Ach-induced relaxations are almost completely inhibited in the presence of L-NAME indicating their mediation by NO. This can be appreciated from the findings shown in S5H and 5J Figure (see also below). We have added this data to the results (page 14, line 298-304).

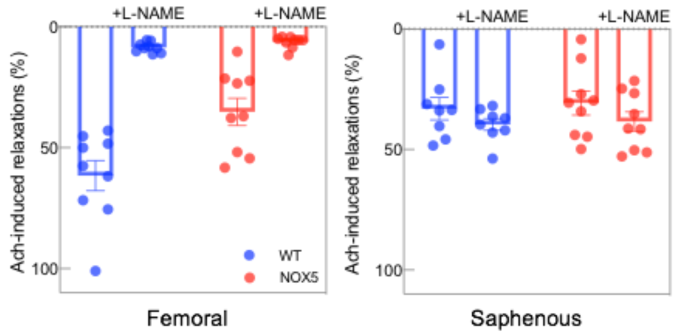

Comment 9: The degree of acetylcholine mediated relaxation in the saphenous artery (figure 3b) seems very impaired. Normally vessels of this size relax at least by 60 to 75%. This is also true for the responses in 3A.

Response: We agree with the reviewer that isolated saphenous arteries of the mouse can display large relaxing responses to Ach. This is the case for young and young adult mice as we have previously reported [29-31]. In the aged control and NOX5 KI mice these responses were, however, comparably attenuated. Another possible reason is the genetic background of mice. In our study we are using mice with mixed background (80% 129/Sv and 20% C57Bl6) and previous data showed that endothelium-dependent-relaxation is smaller in 129/Sv versus C57Bl6 mice [32]. This has been clarified in the manuscript (page 13, line 271-276).

Comment 10: What is the significance of the inhibition of PE contraction by indomethacin? This would suggest a role of some vasoconstrictor prostanoid, but which one? How does this relate to Nox5?

Response: In the thoracic aorta of aged mice, indomethacin indeed markedly reduced contractile responses to PHE and ET-1 but not high K^+ (see S7G-I Figure). Because this (unexpected) effect did not differ considerably between vessels from control and NOX5 KI mice, we did not investigate it any further because it does not seem to be related to NOX5.

Reviewer 2

In this manuscript, the authors show that hypertensive, microalbuminuric human subjects have increased expression of NOX5. Mouse NOX5KI exhibit increases in systolic BP with aging, and treatment of vasculature from these mice vasodilate in response to sepiapterin. The authors conclude that it is NOX5 that is mediating the hypertenison in both humans and mice.

Response: We thank the reviewer for this crisp summary of our work.

Comment 1: The authors should show telemetry BP in mice for at least 5 days and show mean arterial BP (MAP). Does MAP change with aging in NOX5KI mice compared to controls.

Response: We did the telemetry measurement for 3 days and not 5 days as in many previous publications [33-35]. Thus, the telemetry data that we had presented in the manuscript was representative of 72h (3 days). The average per hour was calculated for the 72h period. In the revised manuscript, we have changed the figures to show the telemetry measurements of every hour for 3 days (see Figure 2, S2 Figure and below). This has been also changed in the Methods (page 26, line 589). With respect to MAP, we have added a figure (see S2 Figure and below) which shows the MAP results for both aged and young mice. As we show in the figure, the MAP was significantly higher in aged NOX5KI mice compared to aged WT. This indicates that (as described in the text) the NOX5 KI mice develop a selective elevation of systolic blood pressure. In young mice, there was no difference in MAP between NOX5KI and WT. This has also been added to the results (page 11, line 221-225).

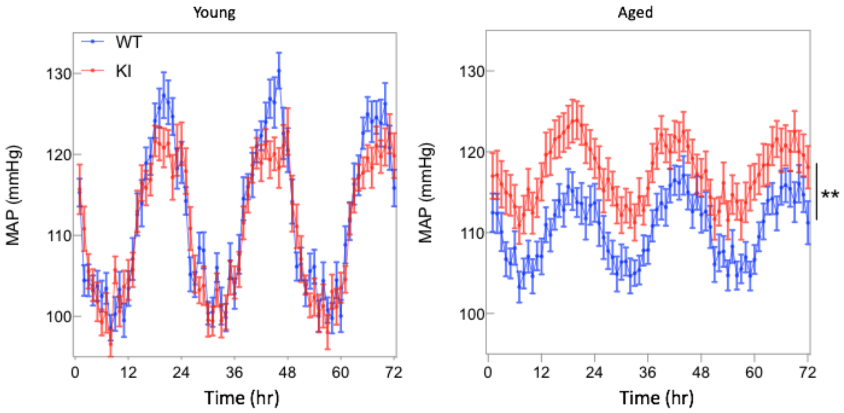

Comment 2: Does treatment of mice with sepiapterin decrease BP in aging NOX5KI mice?

Response: This is a legitimate question. Unfortunately, it cannot be addressed experimentally in a reasonable period of time. The elevation of systolic BP in NOX5 KI mice became apparent when we recorded hemodynamics in mice of progressively increasing age. These mice had to be euthanized in order to isolate their blood vessels and test hypotheses concerning the underlying mechanisms. The size of the colonies of mice is small and it would take many months/years to reach the necessary number of animals of advanced age to do the necessary experiments. This experiment would have strengthened our conclusion about NOX5-induced NOS uncoupling. Indeed, sepiapterin or BH4 were shown in some preclinical and clinical studies to slightly reduce the high blood pressure induced by NOS uncoupling [26-28]. We have included a statement in the "discussion" of our manuscript that refers to this desirable extension of our mechanistic *ex vivo* analysis of impaired endothelium-dependent relaxation (uncoupled eNOS) through this additional set of therapeutic *in vivo* experiments (recoupling eNOS; see page 19, line 436-438).

Reviewer 3

The paper entitled NOX5-induced uncoupling of endothelial NO synthase is a causal mechanism and theragnostic target of an age-related hypertension endotype by Elbatreek et al describes a novel role of the superoxide-generating NOX5 isoform as a cause of increased blood pressure in aged mice overexpressing the human NOX5 isoform (NOX5 KI mice)and finds a correlation of increased NOX5 levels in endothelial microparticles with hypertensive patients as well as a potential mechanism by an in silico interactome approach showing a close interation between NOX5 and nitic oxide signalling using human data. Finally, using an ex-vivo pharmacological approach using vessels purified from NOX5 KI mice, they confirm the connection between NOX5-derived ROS and the uncoupling of endothelial NO synthase by recoupling NOS with sepiapterin
The paper is well-written and the methods are sound. It uses human and mouse in vivo and ex vivo data and provides several novel important findings, in particular, the measurement of circulating microparticular NOX5 as a biomarker in a subpopulation of hypertensive patients, providing valuable tool for stratification of potential future clinical trials. Also the spontaneous hypertensive phenotype in NOX5 KI mice is of great interest for further testing of preclinical drugs. Finally the mechanism described is of great interest-at least in this model.
Altogether, the paper can be recommended for publication if the comments below are answered satisfactorily.

Response: We thank the reviewer for this crisp summary and kind analysis. We have responded satisfactorily to all the comments and incorporated changes to reflect all the suggestions provided by the reviewer.

Major comments:

Comment 1: Several statements in the discussion are very strong and mix the findings from human clinical samples and results obtained with mice overexpressing NOX5, a rather artificial system. For example, the mechanism of uncoupling NO synthase by NOX5 has indeed not be proven in humans. The discussion would benefit from a more balanced interpretation and should also highlight potential pitfalls of the animal model.

Response: This is a legitimate and worthwhile comment and recommendation of the reviewer. We have updated the manuscript with a more balanced discussion. With respect to the example that the reviewer mentioned, we agree that NOX5-induced NOS uncoupling has not been proven in humans and we mention clearly in the discussion that our clinical data are yet still correlative (page 18, line 402). To partially strengthen our conclusion about NOS uncoupling in human hypertension, we provide ADMA levels, a biomarker for NOS uncoupling, in both healthy and hypertensive subjects (see S1D Figure and also below). We show that ADMA levels are higher in hypertensive patients compared to controls which is in agreement with previous findings [36, 37]. However, whether this is induced by NOX5 needs further investigations for example, *ex-vivo* analysis of isolated human blood vessels or an interventional trial with sepiapterin. This has been adapted in the results and discussion (page 9 and 20, line 200-203 and 443-447). Regarding the KI animal model, we have highlighted some potential pitfalls of this model. For example, there are fundamental differences between human and mice physiology and human protein expressed in mice may not wholly reflect its role in human pathology [38]. We have added this to the discussion (page 19-20, line 438-442).

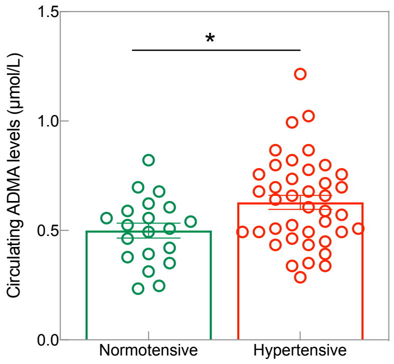

Comment 2: In a previous paper (Casas et al, 2019) describing the NOX5 KI model, the same authors show high expression levels of NOX5 in white blood cells. First, this fact should be documented in the present study and the authors should comment on how they exclude a potential effect of leukocytic NOX5 on hypertension in aged animals as the ex vivo data do not solely exclude this.

Response: As suggested by the reviewer, we have documented that NOX5 is also expressed in white blood cells of the NOX5 KI mice (page 16 and 25, line 359-360 and 561). In our *in vivo* animal experiments, we cannot exclude a potential effect of leukocytic NOX5 on hypertension in aged animals; in our *ex vivo* experiments a contribution of adhering leukocytes is unlikely. Despite this limitation, our NOX5 KI mouse model also mimics, to a large extent, the physiological pattern of NOX5 expression in humans i.e. in endothelial cells and white blood cells [39]. In addition, clinically, this may be a minor limitation because NOX inhibitors will always act systemically and not in a cell-specific manner. This limitation has been addressed in the revised version of the manuscript (page 16, line 359-363).

Comment 3: Addition of antioxidants or the pharmacological tool DPI to the ex vivo experiments would be an asset. Has it be done? Have these compounds been tested? These data should be added whatever the outcome and interpreted accordingly.

Response: Indeed, we have tested the effect of the antioxidants N-acetyl cysteine (NAC) and tempol. Both antioxidants were unable to reverse the impaired endothelium-dependent relaxations in femoral arteries isolated from NOX5 KI mice (see S10 Figure and also below). Possible interpretation of these negative results is that these compounds can scavenge the superoxide produced by NOX5 *ex vivo*, yet they are not able to recouple NOS which had been uncoupled by chronic NOX5-produced ROS exposure *in vivo*. Therefore, the effect of chronic administration of these compounds in NOX5 KI mice needs to be examined. With respect to DPI, using this compound will not give a conclusive results as it is a non-selective general flavoprotein inhibitor [40]. This has been added to the results and discussed in the manuscript (page 15 and 19, line 336-338 and 433-435).

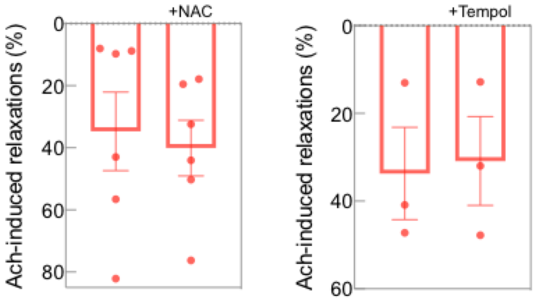

Comment4: NOX5 is known to be expressed in leucocytes. Is it possible that some contaminating white blood cells are present in endothelial microparticles. How was this excluded?

Response: The possibility of presence of white blood cells is very low for three reasons. First, endothelial microparticles was extracted from the plasma and not the whole blood (the protocol was used by us in previous publications [41-44]). Second, endothelial microparticles were isolated using anti-CD144 antibody which targets endothelial cell-specific protein i.e. CD144 (also known as vascular endothelial cadherin) [45, 46]. Third, the purity of CD144+ microparticles was determined by FACS analysis. With the use of FITC-conjugated beads as size references, the size of such particles was assessed to be <0.5 µm in diameter while white blood cells are larger i.e. 5-20µm. This is clarified in the Methods (page 24, line 541-548). Overall, even if there is a contamination of WBCs, this does not affect our conclusions as the same applies for both control and disease groups.

Minor comments:

Comment5: Page 12, figure S4. Page 13, lanes 216-225: document in the text the concentrations used for PAPA/NO, BAY 60-2770 and sepiapterin

Response: The concentrations of PAPA/NO, BAY 60-2770 and sepiapterin have been documented in the text (page 15, line 323, 324 and 332) and relevant figure legends.

Comment6: Page 17, lane 297: Here NOX5 is important….: sentence is not clear. What the "here" refer to?

Response: “Here” refers to endothelial cells. We changed the phrasing of this sentence to more clearly formulate our point of view (page 19, line 426).

Comment7: Page 15-17. What are the chances of administering sepiapterin in patients?

Response: To our knowledge, sepiapterin has not been tested in hypertensive patients. However, folic acid and BH4 were shown in some clinical studies to slightly reduce the high blood pressure by improving endothelial function [27, 47]. Folic acid also reduces the risk of stroke by 73% in a subgroup of hypertensive patients [48]. In addition, combination of folic acid with the antihypertensive enalapril significantly reduced the risk of stroke compared to enalapril alone [49]. A recent metanalysis showed that combination of folic acid with antihypertensive drugs in patients with hypertension is effective in reducing the risk of cardiovascular and cerebrovascular events and lowering blood pressure [50]. It may be worth reconsidering sepiapterin (or its FDA-approved analogue sapropterin) in a new study dedicated to isolated systolic hypertension. We now refer to this perspective in the discussion (page 20, line 447-450).

Comment8: Page 22. It is nowadays standard practice to document the sex of the animals used. Have both genders been used in the study? Was blood pressure similar in males and females? Was there a reason to select one gender over another one?

Response: We already mentioned in the manuscript that both genders of mice were used (page 11, 12 and 25, line 221, 225 and 564). There was no difference in blood pressure between males and females. This has been clarified in the manuscript (page 11, line 231-232).

Reviewer 4

Interactome modularity based analysis is acceptable.

Response: We thank the reviewer for the positive comment and valuable feedback on the manuscript.

Comment 1: Figure is not of sufficiently good resolution. The printout was not readable. Please provide high resolution image.

Response: A figure with a higher resolution has now been included. We have updated the figures and increased the resolution from 300 DPI to 600 DPI. Also, we have increased the font of some text in the figures. We apologize for the potential inconvenience in the original version of the manuscript.

References

1. Oparil S, Acelajado MC, Bakris GL, Berlowitz DR, Cifkova R, Dominiczak AF, et al. Hypertension. Nat Rev Dis Primers. 2018;4:18014. Epub 2018/03/23. doi: 10.1038/nrdp.2018.14. PubMed PMID: 29565029; PubMed Central PMCID: PMCPMC6477925.
2. Yogi A, Mercure C, Touyz J, Callera GE, Montezano AC, Aranha AB, et al. Renal redox-sensitive signaling, but not blood pressure, is attenuated by Nox1 knockout in angiotensin II-dependent chronic hypertension. Hypertension. 2008;51(2):500-6. Epub 2008/01/16. doi: 10.1161/HYPERTENSIONAHA.107.103192. PubMed PMID: 18195161.
3. Holterman CE, Read NC, Kennedy CR. Nox and renal disease. Clin Sci (Lond). 2015;128(8):465-81. Epub 2015/01/30. doi: 10.1042/CS20140361. PubMed PMID: 25630236.
4. Saenz-Medina J, Munoz M, Sanchez A, Rodriguez C, Jorge E, Corbacho C, et al. Nox1-derived oxidative stress as a common pathogenic link between obesity and hyperoxaluria-related kidney injury. Urolithiasis. 2019. Epub 2019/11/07. doi: 10.1007/s00240-019-01170-w. PubMed PMID: 31690958.
5. Munoz M, Lopez-Oliva ME, Rodriguez C, Martinez MP, Saenz-Medina J, Sanchez A, et al. Differential contribution of Nox1, Nox2 and Nox4 to kidney vascular oxidative stress and endothelial dysfunction in obesity. Redox Biol. 2020;28:101330. Epub 2019/09/29. doi: 10.1016/j.redox.2019.101330. PubMed PMID: 31563085; PubMed Central PMCID: PMCPMC6812001.
6. Murdoch CE, Alom-Ruiz SP, Wang M, Zhang M, Walker S, Yu B, et al. Role of endothelial Nox2 NADPH oxidase in angiotensin II-induced hypertension and vasomotor dysfunction. Basic Res Cardiol. 2011;106(4):527-38. Epub 2011/04/30. doi: 10.1007/s00395-011-0179-7. PubMed PMID: 21528437; PubMed Central PMCID: PMCPMC3105229.
7. Sag CM, Schnelle M, Zhang J, Murdoch CE, Kossmann S, Protti A, et al. Distinct Regulatory Effects of Myeloid Cell and Endothelial Cell NAPDH Oxidase 2 on Blood Pressure. Circulation. 2017;135(22):2163-77. Epub 2017/03/17. doi: 10.1161/CIRCULATIONAHA.116.023877. PubMed PMID: 28298457; PubMed Central PMCID: PMCPMC5444427.
8. Ray R, Murdoch CE, Wang M, Santos CX, Zhang M, Alom-Ruiz S, et al. Endothelial Nox4 NADPH oxidase enhances vasodilatation and reduces blood pressure in vivo. Arterioscler Thromb Vasc Biol. 2011;31(6):1368-76. Epub 2011/03/19. doi: 10.1161/ATVBAHA.110.219238. PubMed PMID: 21415386.
9. Kleinschnitz C, Grund H, Wingler K, Armitage ME, Jones E, Mittal M, et al. Post-stroke inhibition of induced NADPH oxidase type 4 prevents oxidative stress and neurodegeneration. PLoS biology. 2010;8(9). Epub 2010/09/30. doi: 10.1371/journal.pbio.1000479. PubMed PMID: 20877715.
10. Bubikat A, De Windt LJ, Zetsche B, Fabritz L, Sickler H, Eckardt D, et al. Local atrial natriuretic peptide signaling prevents hypertensive cardiac hypertrophy in endothelial nitric-oxide synthase-deficient mice. J Biol Chem. 2005;280(22):21594-9. Epub 2005/03/29. doi: 10.1074/jbc.M501103200. PubMed PMID: 15793309.
11. Godecke A, Decking UK, Ding Z, Hirchenhain J, Bidmon HJ, Godecke S, et al. Coronary hemodynamics in endothelial NO synthase knockout mice. Circ Res. 1998;82(2):186-94. Epub 1998/02/19. doi: 10.1161/01.res.82.2.186. PubMed PMID: 9468189.
12. Devereux RB, Pickering TG, Alderman MH, Chien S, Borer JS, Laragh JH. Left ventricular hypertrophy in hypertension. Prevalence and relationship to pathophysiologic variables. Hypertension. 1987;9(2 Pt 2):II53-60. Epub 1987/02/01. doi: 10.1161/01.hyp.9.2_pt_2.ii53. PubMed PMID: 2879790.
13. Park JB, Schiffrin EL. Small artery remodeling is the most prevalent (earliest?) form of target organ damage in mild essential hypertension. J Hypertens. 2001;19(5):921-30. Epub 2001/06/08. doi: 10.1097/00004872-200105000-00013. PubMed PMID: 11393676.
14. Cuspidi C, Sala C, Negri F, Mancia G, Morganti A, Italian Society of H. Prevalence of left-ventricular hypertrophy in hypertension: an updated review of echocardiographic studies. J Hum Hypertens. 2012;26(6):343-9. Epub 2011/11/25. doi: 10.1038/jhh.2011.104. PubMed PMID: 22113443.
15. Bloksgaard M, Leurgans TM, Spronck B, Heusinkveld MHG, Thorsted B, Rosenstand K, et al. Imaging and modeling of acute pressure-induced changes of collagen and elastin microarchitectures in pig and human resistance arteries. Am J Physiol Heart Circ Physiol. 2017;313(1):H164-H78. Epub 2017/04/23. doi: 10.1152/ajpheart.00110.2017. PubMed PMID: 28432057.

16. Pourageaud F, De Mey JG. Structural properties of rat mesenteric small arteries after 4-wk exposure to elevated or reduced blood flow. Am J Physiol. 1997;273(4):H1699-706. Epub 1997/11/15. doi: 10.1152/ajpheart.1997.273.4.H1699. PubMed PMID: 9362233.
17. Bezie Y, Lamaziere JM, Laurent S, Challande P, Cunha RS, Bonnet J, et al. Fibronectin expression and aortic wall elastic modulus in spontaneously hypertensive rats. Arterioscler Thromb Vasc Biol. 1998;18(7):1027-34. Epub 1998/07/22. doi: 10.1161/01.atv.18.7.1027. PubMed PMID: 9672062.
18. Hayoz D, Rutschmann B, Perret F, Niederberger M, Tardy Y, Mooser V, et al. Conduit artery compliance and distensibility are not necessarily reduced in hypertension. Hypertension. 1992;20(1):1-6. Epub 1992/07/01. doi: 10.1161/01.hyp.20.1.1. PubMed PMID: 1618544.
19. Lacolley P, Ghodsi N, Glazer E, Challande P, Brissac AM, Safar ME, et al. Influence of graded changes in vasomotor tone on the carotid arterial mechanics in live spontaneously hypertensive rats. Br J Pharmacol. 1995;115(7):1235-44. Epub 1995/08/01. doi: 10.1111/j.1476-5381.1995.tb15031.x. PubMed PMID: 7582551; PubMed Central PMCID: PMCPMC1908801.
20. Intengan HD, Schiffrin EL. Structure and mechanical properties of resistance arteries in hypertension: role of adhesion molecules and extracellular matrix determinants. Hypertension. 2000;36(3):312-8. Epub 2000/09/16. doi: 10.1161/01.hyp.36.3.312. PubMed PMID: 10988257.
21. Bussy C, Boutouyrie P, Lacolley P, Challande P, Laurent S. Intrinsic stiffness of the carotid arterial wall material in essential hypertensives. Hypertension. 2000;35(5):1049-54. Epub 2000/05/20. doi: 10.1161/01.hyp.35.5.1049. PubMed PMID: 10818063.
22. Laurent S, Girerd X, Mourad JJ, Lacolley P, Beck L, Boutouyrie P, et al. Elastic modulus of the radial artery wall material is not increased in patients with essential hypertension. Arterioscler Thromb. 1994;14(7):1223-31. Epub 1994/07/01. doi: 10.1161/01.atv.14.7.1223. PubMed PMID: 8018679.
23. Laurent S, Hayoz D, Trazzi S, Boutouyrie P, Waeber B, Omboni S, et al. Isobaric compliance of the radial artery is increased in patients with essential hypertension. J Hypertens. 1993;11(1):89-98. Epub 1993/01/01. doi: 10.1097/00004872-199301000-00013. PubMed PMID: 8382244.
24. Laurent S. Arterial wall hypertrophy and stiffness in essential hypertensive patients. Hypertension. 1995;26(2):355-62. Epub 1995/08/01. doi: 10.1161/01.hyp.26.2.355. PubMed PMID: 7635546.
25. Gebhart V, Reiss K, Kollau A, Mayer B, Gorren ACF. Site and mechanism of uncoupling of nitric-oxide synthase: Uncoupling by monomerization and other misconceptions. Nitric Oxide. 2019;89:14-21. Epub 2019/04/26. doi: 10.1016/j.niox.2019.04.007. PubMed PMID: 31022534.
26. Wang S, Xu J, Song P, Wu Y, Zhang J, Chul Choi H, et al. Acute inhibition of guanosine triphosphate cyclohydrolase 1 uncouples endothelial nitric oxide synthase and elevates blood pressure. Hypertension. 2008;52(3):484-90. Epub 2008/07/23. doi: 10.1161/HYPERTENSIONAHA.108.112094. PubMed PMID: 18645049; PubMed Central PMCID: PMCPMC3523107.
27. Porkert M, Sher S, Reddy U, Cheema F, Niessner C, Kolm P, et al. Tetrahydrobiopterin: a novel antihypertensive therapy. J Hum Hypertens. 2008;22(6):401-7. Epub 2008/03/07. doi: 10.1038/sj.jhh.1002329. PubMed PMID: 18322548.
28. Podjarny E, Hasdan G, Bernheim J, Rashid G, Green J, Korzets Z, et al. Effect of chronic tetrahydrobiopterin supplementation on blood pressure and proteinuria in 5/6 nephrectomized rats. Nephrol Dial Transplant. 2004;19(9):2223-7. Epub 2004/07/15. doi: 10.1093/ndt/gfh383. PubMed PMID: 15252157.
29. Chennupati R, Lamers WH, Koehler SE, De Mey JG. Endothelium-dependent hyperpolarization-related relaxations diminish with age in murine saphenous arteries of both sexes. Br J Pharmacol. 2013;169(7):1486-99. Epub 2013/03/16. doi: 10.1111/bph.12175. PubMed PMID: 23488619; PubMed Central PMCID: PMCPMC3724106.
30. Chennupati R, Meens MJ, Marion V, Janssen BJ, Lamers WH, De Mey JG, et al. Endothelial arginine resynthesis contributes to the maintenance of vasomotor function in male diabetic mice. PLoS One. 2014;9(7):e102264. Epub 2014/07/18. doi: 10.1371/journal.pone.0102264. PubMed PMID: 25033204; PubMed Central PMCID: PMCPMC4102520.
31. Chennupati R, Meens MJ, Janssen BJ, van Dijk P, Hakvoort TBM, Lamers WH, et al. Deletion of endothelial arginase 1 does not improve vasomotor function in diabetic mice. Physiol Rep. 2018;6(11):e13717. Epub 2018/06/12. doi: 10.14814/phy2.13717. PubMed PMID: 29890043; PubMed Central PMCID: PMCPMC5995309.

32. Ryan MJ, Didion SP, Davis DR, Faraci FM, Sigmund CD. Endothelial dysfunction and blood pressure variability in selected inbred mouse strains. Arterioscler Thromb Vasc Biol. 2002;22(1):42-8. Epub 2002/01/15. doi: 10.1161/hq0102.101098. PubMed PMID: 11788459.
33. Wang Y, Thorin E, Luo H, Tremblay J, Lavoie JL, Wu Z, et al. EPHB4 Protein Expression in Vascular Smooth Muscle Cells Regulates Their Contractility, and EPHB4 Deletion Leads to Hypotension in Mice. J Biol Chem. 2015;290(22):14235-44. Epub 2015/04/24. doi: 10.1074/jbc.M114.621615. PubMed PMID: 25903126; PubMed Central PMCID: PMCPMC4447992.
34. Xu P, Costa-Goncalves AC, Todiras M, Rabelo LA, Sampaio WO, Moura MM, et al. Endothelial dysfunction and elevated blood pressure in MAS gene-deleted mice. Hypertension. 2008;51(2):574-80. Epub 2008/01/09. doi: 10.1161/HYPERTENSIONAHA.107.102764. PubMed PMID: 18180400.
35. Shirey-Rice JK, Klar R, Fentress HM, Redmon SN, Sabb TR, Krueger JJ, et al. Norepinephrine transporter variant A457P knock-in mice display key features of human postural orthostatic tachycardia syndrome. Dis Model Mech. 2013;6(4):1001-11. Epub 2013/04/13. doi: 10.1242/dmm.012203. PubMed PMID: 23580201; PubMed Central PMCID: PMCPMC3701219.
36. Perticone F, Sciacqua A, Maio R, Perticone M, Maas R, Boger RH, et al. Asymmetric dimethylarginine, L-arginine, and endothelial dysfunction in essential hypertension. J Am Coll Cardiol. 2005;46(3):518-23. Epub 2005/08/02. doi: 10.1016/j.jacc.2005.04.040. PubMed PMID: 16053968.
37. Sonmez A, Celebi G, Erdem G, Tapan S, Genc H, Tasci I, et al. Plasma apelin and ADMA Levels in patients with essential hypertension. Clin Exp Hypertens. 2010;32(3):179-83. Epub 2010/05/28. doi: 10.3109/10641960903254505. PubMed PMID: 20504125.
38. Sundberg JP, Berndt A, Sundberg BA, Silva KA, Kennedy V, Bronson R, et al. The mouse as a model for understanding chronic diseases of aging: the histopathologic basis of aging in inbred mice. Pathobiol Aging Age Relat Dis. 2011;1. Epub 2011/01/01. doi: 10.3402/pba.v1i0.7179. PubMed PMID: 22953031; PubMed Central PMCID: PMCPMC3417678.
39. Casas AI, Kleikers PW, Geuss E, Langhauser F, Adler T, Busch DH, et al. Calcium-dependent blood-brain barrier breakdown by NOX5 limits postreperfusion benefit in stroke. J Clin Invest. 2019;130:1772-8. Epub 2019/03/19. doi: 10.1172/JCI124283. PubMed PMID: 30882367; PubMed Central PMCID: PMCPMC6436900.
40. Altenhofer S, Radermacher KA, Kleikers PW, Wingler K, Schmidt HH. Evolution of NADPH Oxidase Inhibitors: Selectivity and Mechanisms for Target Engagement. Antioxid Redox Signal. 2015;23(5):406-27. doi: 10.1089/ars.2013.5814. PubMed PMID: 24383718; PubMed Central PMCID: PMCPMC4543484.
41. Chen Z, Wen L, Martin M, Hsu CY, Fang L, Lin FM, et al. Oxidative stress activates endothelial innate immunity via sterol regulatory element binding protein 2 (SREBP2) transactivation of microRNA-92a. Circulation. 2015;131(9):805-14. Epub 2015/01/01. doi: 10.1161/CIRCULATIONAHA.114.013675. PubMed PMID: 25550450; PubMed Central PMCID: PMCPMC4351177.
42. Hsu CY, Huang PH, Chiang CH, Leu HB, Huang CC, Chen JW, et al. Increased circulating endothelial apoptotic microparticle to endothelial progenitor cell ratio is associated with subsequent decline in glomerular filtration rate in hypertensive patients. PLoS One. 2013;8(7):e68644. Epub 2013/07/23. doi: 10.1371/journal.pone.0068644. PubMed PMID: 23874701; PubMed Central PMCID: PMCPMC3709900.
43. Huang PH, Huang SS, Chen YH, Lin CP, Chiang KH, Chen JS, et al. Increased circulating CD31+/annexin V+ apoptotic microparticles and decreased circulating endothelial progenitor cell levels in hypertensive patients with microalbuminuria. J Hypertens. 2010;28(8):1655-65. Epub 2010/06/04. doi: 10.1097/HJH.0b013e32833a4d0a. PubMed PMID: 20520578.
44. Shang F, Wang SC, Hsu CY, Miao Y, Martin M, Yin Y, et al. MicroRNA-92a Mediates Endothelial Dysfunction in CKD. J Am Soc Nephrol. 2017;28(11):3251-61. Epub 2017/07/12. doi: 10.1681/ASN.2016111215. PubMed PMID: 28696247; PubMed Central PMCID: PMCPMC5661278.
45. Lampugnani MG, Resnati M, Raiteri M, Pigott R, Pisacane A, Houen G, et al. A novel endothelial-specific membrane protein is a marker of cell-cell contacts. J Cell Biol. 1992;118(6):1511-22. Epub 1992/09/01. doi: 10.1083/jcb.118.6.1511. PubMed PMID: 1522121; PubMed Central PMCID: PMCPMC2289607.
46. Breviario F, Caveda L, Corada M, Martin-Padura I, Navarro P, Golay J, et al. Functional properties of human vascular endothelial cadherin (7B4/cadherin-5), an endothelium-specific cadherin.

Arterioscler Thromb Vasc Biol. 1995;15(8):1229-39. Epub 1995/08/01. doi: 10.1161/01.atv.15.8.1229. PubMed PMID: 7627717.
47. McRae MP. High-dose folic acid supplementation effects on endothelial function and blood pressure in hypertensive patients: a meta-analysis of randomized controlled clinical trials. J Chiropr Med. 2009;8(1):15-24. Epub 2009/08/04. doi: 10.1016/j.jcm.2008.09.001. PubMed PMID: 19646382; PubMed Central PMCID: PMCPMC2697578.
48. Kong X, Huang X, Zhao M, Xu B, Xu R, Song Y, et al. Platelet Count Affects Efficacy of Folic Acid in Preventing First Stroke. J Am Coll Cardiol. 2018;71(19):2136-46. Epub 2018/05/12. doi: 10.1016/j.jacc.2018.02.072. PubMed PMID: 29747834.
49. Huo Y, Li J, Qin X, Huang Y, Wang X, Gottesman RF, et al. Efficacy of folic acid therapy in primary prevention of stroke among adults with hypertension in China: the CSPPT randomized clinical trial. JAMA. 2015;313(13):1325-35. Epub 2015/03/17. doi: 10.1001/jama.2015.2274. PubMed PMID: 25771069.
50. Wang WW, Wang XS, Zhang ZR, He JC, Xie CL. A Meta-Analysis of Folic Acid in Combination with Anti-Hypertension Drugs in Patients with Hypertension and Hyperhomocysteinemia. Front Pharmacol. 2017;8:585. Epub 2017/09/16. doi: 10.3389/fphar.2017.00585. PubMed PMID: 28912716; PubMed Central PMCID: PMCPMC5584015.
