# Supplementary material for: NOX5-induced uncoupling of endothelial NO synthase is a causal mechanism and theragnostic target of an age-related hypertension endotype
Source: PLoS Biol. 2020 Nov 10;18(11):e3000885. doi: 10.1371/journal.pbio.3000885 (PMC7654809; doi:10.1371/journal.pbio.3000885)
Supplement: Attachment — Submitted filename: Response to Reviewers comments_v3.pdf [file pbio.3000885.s018.pdf]

POINT-BY-POINT RESPONSE TO THE EDITOR AND REVIEWERS

To the Editor

ETHICS STATEMENT:

Please indicate within your manuscript if your human experiments approved by the research ethics committee of Taipei Veterans General Hospital adhered to the Declaration of Helsinki or any other specific national or international ethical guidelines.

Response: We have indicated that the human experiments approved by the research ethics committee of Taipei Veterans General Hospital are in adherence to the Declaration of Helsinki.

Please include the full name of the IACUC/ethics committee that reviewed and approved the animal care and use protocol/permit/project license. Please also include an approval number.

Response: All animal experimental research was approved by the Animal Welfare Body (IvD) of Maastricht University, the Animal Ethics Committee (Dier Experimenten Commissie, DEC) and the Central Authority for Scientific Procedures on Animals (Centrale Commissie Dierproeven, CCD). The DEC project numbers are 2012-123 and PV 2017-029, and license number is AVD1070020174486.

Please include the specific national or international regulations/guidelines to which your animal care and use protocol adhered. Please note that institutional or accreditation organization guidelines (such as AAALAC) do not meet this requirement.

Response: The animal care and use protocol adhere to the ARRIVE guidelines (Animal Research: Reporting of In Vivo Experiments), https://arriveguidelines.org/, Wet op dierproeven (WoD, 2014, https://wetten.overheid.nl/BWBR0003081/2019-01-01), the Dierproevenregeling (DpR, 2014, https://zoek.officielebekendmakingen.nl/stcrt-2014-34746.html), the Dierproevenbesluit (DpB, 2014, https://wetten.overheid.nl/BWBR0035866/2014-12-18), and the EU Directive 2010/63 (2010, https://eur-lex.europa.eu/eli/dir/2010/63/2019-06-26)

DATA POLICY:

Please update your S1 Data File to provide the quantitative summary data for Fig 1CD.

Response: We have updated the S1 Data File to provide the quantitative summary data for Fig 1C, D.

Please ensure that each figure legend in your manuscript include information on where the underlying data can be found and ensure your supplemental data file/s has a legend.

Response: We have ensured that each figure legend in our manuscript includes information on where the underlying data can be found and ensured that all supplemental data file/s have a legend. According to the journal guidelines, supplementary legends are present at the end of the manuscript file.

To reviewer 1

Comment 1: The authors have responded to prior concerns. There continue to be a few statements that are incorrect that could be easily modified.

Response: We thank the reviewer for the valuable comments. We have modified the statements that the reviewer refers to, i.e. the role of NOX1 in preclinical hypertension and discussed the hypertensive phenotype observed upon ageing of mice overexpressing p22phox or lacking the vascular ecSOD.

Comment 2: It is not true that Nox1 has not been shown to have a role in pre-clinical studies of hypertension. Dikalova et al showed that overexpression of this isoform potentiated hypertension in mice (PMID 16230485). Gawazzi et al showed that Nox1 deficient mice had a complete loss of the sustained hypertensive response to angiotensin II.

Response: Indeed, we agree with the reviewer. The paper by Dikalova *et al* [1] that the reviewer quotes is the work of one of the coauthors (HHHW Schmidt). In this paper, overexpression of NOX1 did not lead to increase in blood pressure unless the mice were infused with angiotensin II for two weeks. This might indicate that NOX1 plays a non-causal role in hypertension. The second paper by Gavazzi *et al* [2] indeed showed that Nox1 deficient mice had a complete loss of the sustained hypertensive response to angiotensin II, but also showed that the basal blood pressure is decreased in these mice which indicates a role of NOX1 in regulating basal blood pressure. The paper by Yogi *et al* [3] that we quote in the manuscript shows that NOX1 plays no role in chronic angiotensin II-dependent hypertension. Overall, the data regarding the role of NOX1 regarding preclinical hypertension are controversial. This point has been clarified in the manuscript (Page 5-6, Lines 65-71)

Comment 3: The authors should note that the hypertensive response with aging in the Nox5 ki mice is very similar to that observed in mice overexpressing p22phox in the vascular smooth muscle and in mice lacking the vascular smooth muscle ecSOD (see PMID 26595812).

Response: We thank the reviewer for this valuable addition. p22phox is an essential component of NOX 1-4 and the paper quoted by the reviewer [4] shows that mice overexpressing this protein develop hypertension upon ageing and this is attenuated by treatment with antioxidants. This paper also shows that deletion of the antioxidant ecSOD from vascular smooth muscle cells leads to hypertension upon ageing. This has been added to the manuscript (Page 5, Lines 67-69).

To reviewer 2

In this manuscript the authors provide evaluation of NOX5 and NOS3 based on human interactome analyses. They go on to develop a mouse NOX5 gene knock in into endothelial and WBCs. They find that the aging NOX5 KI mice are hypertensive, but not the young adult animals. They claim that it is the muscular conduit vessels that mediate the hypertension in the aging KI. The manuscript takes many liberties with the analyses of previous studies that are not quite correct.

Response: We thank the reviewer for the crisp summary for our manuscript. We thoroughly addressed all of the reviewer's comments/questions throughout the entire manuscript, in particular with respect to sections referred to as 'liberties', i.e., the role of NOX1 and NOX2 in hypertension, and the role of antioxidants in other hypertensive models where NOX5 is missing.

Comment 1: They also interpret data to show the importance of conduit vessels (lines 198-99) without ever measuring endothelial function in resistance vessels, particularly in the kidney that are known to control sodium handling and hypertension.

Response: In our study, we have analyzed the structural, smooth muscle and endothelial vasomotor properties of one of the small muscular resistance arteries i.e., saphenous artery (lumen diameter <250 µm, widely accepted to represent their resistance-sized nature). However, we have not considered other small resistance arteries including renal arteries which control sodium handling and blood pressure [5]. This limitation has been discussed in the revised manuscript (Page 20, Lines 392-394). However, we wish to emphasize that our model represents essential systolic hypertension rather than the renovascular secondary hypertension.

Comment 2: Furthermore, they do not see antioxidants, tempol or N-acetyl cysteine, having any effect on vascular function/hypertension in the KI mice. The lack of antioxidant protection against the hypertension suggests that there is no NO being produced likely due to age-related endothelial dysfunction to cause a response when the ROS are removed.

Response: We agree with the reviewer as we mentioned in the discussion that the endothelium-dependent impaired relaxation in the aged KI mice was not reversed in our ex vivo experiments by antioxidants, presumably because NOS uncoupling was already established chronically in vivo by NOX5-derived superoxide. We now also add that the lack of antioxidant protection against the impaired relaxation suggests that there is may be no longer NO produced possibly as part of age-related endothelial dysfunction (Page 20, Lines 396-397).

Comment 3: The authors discuss the role played by H2O2 as a vasodilator, without mentioning peroxynitrite which is a much better vasodilator.

Response: We agree with the reviewer that peroxynitrite could act as a vasodilator probably by formation of nitric oxide and nitrosothiols [6, 7]. We have discussed the vasodilator effect of peroxynitrite in the revised manuscript (Page 19-20, Lines 377-378).

Comment 4: "eNOS KO mice do not have cardiac hypertrophy". Whole body eNOS KO is developmentally lethal, isn't it?

Response: We do not disagree with the reviewer. We have mentioned that the hypertensive eNOS KO mice do not have cardiac hypertrophy in the context to show that - similar to our aged NOX5 KI model - hypertension does not necessarily coexist with cardiac hypertrophy.

Comment 5: "no difference in BP between males and females". The data should be shown since there are only 9 mice of both sexes that were used for MAP.

Response: We now show the data for MAP of 72 h in aged KI males (120 ± 3.4, n=15) and females (114 ± 2.1, n=18). There was no significant difference between them (P=0.13) (Page 12, Lines 199). The number of aged mice used in MAP in total are 31 and 33 for WT and KI, respectively and not 9 as written in the legend for S2 Figure.

Comment 6: In the human studies, why were so few women included?

Response: The human samples were collected from Taipei Veterans General Hospital, which serves mainly middle-aged and elderly male patients. In addition, most of the patients in our study are below 65 years old and the prevalence of hypertension at this age is higher in males than females [8].

Comment 7: The authors mention that NOX1 and NOX2 do not cause hypertension unless prohypertensive drugs are given (lines 65-67)-antioxidants reduce BP in SHR and Dahl salt sensitive rats, that do not have NOX5.

Response: We suggest that NOX5-related hypertension is one mechanistic endotype and cannot explain all endotypes of hypertension. For that, we analyzed a humanized KI mouse model that expresses NOX5 in its physiological endothelial and WBCs locations. NOX1 and NOX2 on their own do not cause hypertension. Deletion of NOX1 or NOX2 leads to decrease in basal blood pressure which may indicate that these isoforms contribute to regulation of normal blood pressure [2, 9]. Overexpression of these isoforms does not cause elevation of blood pressure unless the animals are infused by angiotensin II [1, 10] which may indicate that NOX1 and NOX2 do not play causal roles in hypertension. This has been clarified in the manuscript (Page 5-6, Lines 65-71).

With respect to SHR and Dahl salt sensitive rats, first we emphasize that the hypertensive phenotype in these models may represent other endotypes where NOX5 is not involved. Regarding the decrease in blood pressure by antioxidants in these models [11-13] this could suggest that in other hypertensive endotypes other ROS sources (mitochondria, xanthine oxidase or myeloperoxidase) play a role. However, the use of these antioxidants cannot indicate a role for any specific ROS source. This has been discussed in the revised manuscript (Page 20, Lines 383-387).

Comment 8: The figures as shown in the manuscript are not of publication quality.

Response: We have used figures with the maximum resolution recommended by the journal (600 dpi). These figures have passed the quality check by the journal before sending to the reviewers. In addition, we have downloaded the figures from the pdf composed by the journal, and they were pretty clear. We think that the reviewer had looked at the figures in the pdf and did not download the high-resolution TIFF file from the blue link on the upper right side of each figure.

To reviewer 3

The authors replied to the comments satisfactorily

Response: We thank the reviewer for the positive feedback.

<u>To reviewer 5</u>

This paper investigates the causal link between NOX5 gene in humans and age-related pulmonary hypertension via uncoupling of endothelial NO synthase. The study starts with computational prediction of PPI networks, proceeds with analysis of control and PH patients and ends with mouse knock-in experiments (mice do not have endogenous NOX5 gene). I found the mouse results convincing, although I cannot say the same for the human results for the following reasons.

Response: We thank the reviewer for the crisp summary for our manuscript. We thoroughly addressed all of the reviewer's comments/questions throughout the entire manuscript. Clearly the mechanistic focus and strongest evidence lays in the mice data; the human data provide an exciting clinical lead, which we are now planning on following up in a major H2020-funded multicenter, multinational clinical trial (HYPERNET). Naturally, only this will be able to provide the ultimate clinical evidence and therapeutic relevance.

Comment 1: The human IID database cannot map three of the seed proteins (GUCYA1, GUCYA2, GUCYB1) and reports no experimentally validated PPIs for NOX3. So, it is strange that the identified "NOX5 subnetwork; Fig 1) includes both GUCYA1 and GUCYB1. Is it a nomenclature issue? If so, the correct gene names need to be reported in the paper. If not, an explanation is warranted. Is it that they did not look for networks with experimental support? If so, they should correct the manuscript to reflect the actual search they performed.

Response: Indeed, we have used only 11 seed proteins extracted a molecular subnetwork from experimentally validated protein-protein interactions 400 from the IID [14] database (interactome) version 2018-11. We did not use NOX3 as a seed protein as this isoform is not expressed in the vasculature, but only in the inner ear [15] and that is why it did not appear in the network figure. We apologize for this unintended mistake and have deleted NOX3 from the seed proteins mentioned in the manuscript (Page 6, 23, Lines 89, 452). With respect to GCY proteins, they must appear together as these constitute the subunits of one enzyme, soluble guanylate cyclase. The names of the proteins have been corrected in the manuscript (Page 6, 23, Lines 89, 453) and Figure 1 from GUCYA1, GUCYA2 and GUCYB1 (gene names) to GCYA1, GCYA2 and GCYB1 (protein names), respectively.

Comment 2: Furthermore, NOX5 had only one study to support its PPIs. The authors should discuss the limitations of their predictive model that is based on such limited evidence. Subsequently any claims about "causality" (in the sense of direct causal effects) should be toned down as they may constitute an overstatement of the results.

Response: We agree with the reviewer that only one study showed a physical interaction between NOX5 and soluble guanylate cyclase as suggested by high-throughput affinity chromatography [16]. We now refer to this as a potential limitation in the discussion (Page 18, Lines 349-351). Our claims about causality stem not only from the PPIs data, but also from our clinical and preclinical findings. In particular in the mouse model as mice that express NOX5 develop hypertension spontaneously without any intervention.

Comment 3: Fig 1 is badly presented, and it is unclear if it supports the authors' claims. First the resolution makes the axes labels unreadable. We can only guess what the authors want to say. Second, Fig 1C and 1D should probably be reversed, because one first identifies the bimodal distribution among hypertension patients and then compares the groups (unless the groups were identified with external characteristics, like albuminuria). Third, the lines with the asterisks in fig. 1C do not extend to make

clear what they compare these groups to. Are they compared to the controls? Fourth the actual p-values should be provided after correction. From the bi-modal distribution presentation in Fig. 1D it looks like the first group of hypertension patients may not be so different than controls.

Response: First, we have used figures with the maximum resolution recommended by the journal (600 dpi). These figures have passed the quality check by the journal before sending to the reviewers. In addition, we have downloaded the figures from the pdf composed by the journal, and they were pretty clear, and all axes labels were readable. We think that the reviewer had looked at the figures in the pdf and did not download the high-resolution TIFF file from the blue link on the upper right side of each figure.

Second, we cannot reverse Fig 1C and 1D as the groups were identified with external characteristics, i.e., albuminuria. Therefore, we first compared NOX5 levels between groups then we performed the subgroup analysis.

Third, the asterisks in fig. 1C refer to comparison to control healthy subjects. We have made this clear in the revised figure by extending the lines.

Fourth, we now provide the actual p values in the legend of Figure 1.

Fifth, the reviewer's comment about Fig 1D is correct and in agreement with our conclusion. In Fig 1D, and as we clarify in the text and the legend, we did a subgroup analysis of all hypertensive patients (with and without microalbuminuria), therefore, we could see that only around 25% of all the hypertensive patients have high NOX5 levels compared to controls. The first group of hypertensive patients (the left red peak) is not significantly different from the controls (the green peak) ($p = 0.49$). We have clarified this in the figure legend. We also wish to emphasize as we mention on the discussion that our clinical data, though significant, is limited by the relatively small number of patients in the present study and needs to be validated in larger and international hypertension cohort (Page 17, Lines 325-327).

Comment 4: I wish I could clearly read Fig. 1 which is very important and especially fig 1D. What is the Y-axis? The labels are unreadable, and the legend/text does not explain it clearly either. And this is supposed to be a very important evidence because they use the bimodal distribution to split the hypertensive patients into two groups. If what they measure is circulating NOX5 from endothelial cells then the first group may be not significantly different from the normal.

Response: Regarding the figure resolution, please see our response to comment 3. The Y-axis in Fig 1D is "Relative frequency (percentage)" which is the most common y-axes label in any frequency distribution analysis, and it is clearly readable in the high-resolution TIFF file. Circulating NOX5 levels (pg/ml) are presented in the X-axis of this figure and not the Y-axis. Again, we agree with the reviewer that the first group of hypertensive patients (the left red peak) is not significantly different from the controls (the green peak). This is what we exactly conclude, that NOX5 levels are higher only on a subgroup of all hypertensives (around 25%) representing an endotype.

Comment 5: The control patients include no smokers and no diabetics. Smoking actually represents a significant difference between the two cohorts, but it is not discussed at all. It is well known that smoking impairs NO synthase and especially in endothelial cells. E.g.:
https://www.ahajournals.org/doi/full/10.1161/01.atv.16.4.546
https://www.atsjournals.org/doi/full/10.1165/ajrcmb.19.5.3091

Response: We agree with the authors that smoking impairs NO synthase and especially in endothelial cells [17]. This effect is now discussed in the revised manuscript (Page 17, Lines 327-328). However, our main point is about different NOX5 levels in a mechanistically defined subgroup.

Comment 6: Beta-blockers and a number of other molecules are differing significantly between the three groups and should be discussed as more direct causes (or results) of the observed differences.

Response: The effect of antihypertensive drugs on NOX5 levels definitely warrants further investigation. We have added this to the revised manuscript as an outlook (Page 17, Lines 327-328).

Comment 7: So overall, I would say this is a very good mouse study but the importance of these results in humans is something that requires further investigation.

Response: We fully agree with the reviewer that the importance of our results in humans is something that requires and deserves extensive further investigation and we have clarified this in the manuscript (Page 17-19, Lines 325-327, 352, 368-370). As mentioned above already, we are now planning on following up on our findings in a major H2020-funded multicenter, multinational clinical trial (HYPERNET).

References

1. Dikalov SI, Dikalova AE, Bikineyeva AT, Schmidt HH, Harrison DG, Griendling KK. Distinct roles of Nox1 and Nox4 in basal and angiotensin II-stimulated superoxide and hydrogen peroxide production. Free Radic Biol Med. 2008;45(9):1340-51. Epub 2008/09/02. doi: 10.1016/j.freeradbiomed.2008.08.013. PubMed PMID: 18760347; PubMed Central PMCID: PMCPMC2630771.
2. Gavazzi G, Banfi B, Deffert C, Fiette L, Schappi M, Herrmann F, et al. Decreased blood pressure in NOX1-deficient mice. FEBS Lett. 2006;580(2):497-504. Epub 2006/01/03. doi: 10.1016/j.febslet.2005.12.049. PubMed PMID: 16386251.
3. Yogi A, Mercure C, Touyz J, Callera GE, Montezano AC, Aranha AB, et al. Renal redox-sensitive signaling, but not blood pressure, is attenuated by Nox1 knockout in angiotensin II-dependent chronic hypertension. Hypertension. 2008;51(2):500-6. Epub 2008/01/16. doi: 10.1161/HYPERTENSIONAHA.107.103192. PubMed PMID: 18195161.
4. Wu J, Saleh MA, Kirabo A, Itani HA, Montaniel KR, Xiao L, et al. Immune activation caused by vascular oxidation promotes fibrosis and hypertension. J Clin Invest. 2016;126(1):50-67. Epub 2015/11/26. doi: 10.1172/JCI80761. PubMed PMID: 26595812; PubMed Central PMCID: PMCPMC4701546.
5. Miksche LW, Miksche U, Gross F. Effect of sodium restriction on renal hypertension and on renin activity in the rat. Circ Res. 1970;27(6):973-84. Epub 1970/12/01. doi: 10.1161/01.res.27.6.973. PubMed PMID: 5487082.
6. Moro MA, Darley-Usmar VM, Lizasoain I, Su Y, Knowles RG, Radomski MW, et al. The formation of nitric oxide donors from peroxynitrite. Br J Pharmacol. 1995;116(3):1999-2004. Epub 1995/10/01. doi: 10.1111/j.1476-5381.1995.tb16404.x. PubMed PMID: 8640338; PubMed Central PMCID: PMCPMC1908945.
7. Wu M, Pritchard KA, Jr., Kaminski PM, Fayngersh RP, Hintze TH, Wolin MS. Involvement of nitric oxide and nitrosothiols in relaxation of pulmonary arteries to peroxynitrite. Am J Physiol. 1994;266(5 Pt 2):H2108-13. Epub 1994/05/11. doi: 10.1152/ajpheart.1994.266.5.H2108. PubMed PMID: 8203609.
8. Ramirez LA, Sullivan JC. Sex Differences in Hypertension: Where We Have Been and Where We Are Going. Am J Hypertens. 2018;31(12):1247-54. Epub 2018/10/10. doi: 10.1093/ajh/hpy148. PubMed PMID: 30299518; PubMed Central PMCID: PMCPMC6233684.
9. Sag CM, Schnelle M, Zhang J, Murdoch CE, Kossmann S, Protti A, et al. Distinct Regulatory Effects of Myeloid Cell and Endothelial Cell NAPDH Oxidase 2 on Blood Pressure. Circulation. 2017;135(22):2163-77. Epub 2017/03/17. doi: 10.1161/CIRCULATIONAHA.116.023877. PubMed PMID: 28298457; PubMed Central PMCID: PMCPMC5444427.
10. Murdoch CE, Alom-Ruiz SP, Wang M, Zhang M, Walker S, Yu B, et al. Role of endothelial Nox2 NADPH oxidase in angiotensin II-induced hypertension and vasomotor dysfunction. Basic Res Cardiol. 2011;106(4):527-38. Epub 2011/04/30. doi: 10.1007/s00395-011-0179-7. PubMed PMID: 21528437; PubMed Central PMCID: PMCPMC3105229.
11. Rodriguez-Iturbe B, Zhan CD, Quiroz Y, Sindhu RK, Vaziri ND. Antioxidant-rich diet relieves hypertension and reduces renal immune infiltration in spontaneously hypertensive rats. Hypertension. 2003;41(2):341-6. Epub 2003/02/08. doi: 10.1161/01.hyp.0000052833.20759.64. PubMed PMID: 12574105.
12. Akpaffiong MJ, Taylor AA. Antihypertensive and vasodilator actions of antioxidants in spontaneously hypertensive rats. Am J Hypertens. 1998;11(12):1450-60. Epub 1999/01/08. doi: 10.1016/s0895-7061(98)00183-6. PubMed PMID: 9880127.
13. Meng S, Cason GW, Gannon AW, Racusen LC, Manning RD, Jr. Oxidative stress in Dahl salt-sensitive hypertension. Hypertension. 2003;41(6):1346-52. Epub 2003/04/30. doi: 10.1161/01.HYP.0000070028.99408.E8. PubMed PMID: 12719439.
14. Kotlyar M, Pastrello C, Malik Z, Jurisica I. IID 2018 update: context-specific physical protein-protein interactions in human, model organisms and domesticated species. Nucleic Acids Res. 2019;47(D1):D581-D9. Epub 2018/11/09. doi: 10.1093/nar/gky1037. PubMed PMID: 30407591; PubMed Central PMCID: PMCPMC6323934.
15. Banfi B, Malgrange B, Knisz J, Steger K, Dubois-Dauphin M, Krause KH. NOX3, a superoxide-generating NADPH oxidase of the inner ear. J Biol Chem. 2004;279(44):46065-72. Epub 2004/08/25. doi: 10.1074/jbc.M403046200. PubMed PMID: 15326186.
16. Huttlin EL, Bruckner RJ, Paulo JA, Cannon JR, Ting L, Baltier K, et al. Architecture of the human interactome defines protein communities and disease networks. Nature. 2017;545(7655):505-9. Epub 2017/05/18. doi: 10.1038/nature22366. PubMed PMID: 28514442; PubMed Central PMCID: PMCPMC5531611.
17. Su Y, Han W, Giraldo C, De Li Y, Block ER. Effect of cigarette smoke extract on nitric oxide synthase in pulmonary artery endothelial cells. Am J Respir Cell Mol Biol. 1998;19(5):819-25. Epub 1998/11/07. doi: 10.1165/ajrcmb.19.5.3091. PubMed PMID: 9806747.
